# Evolution of the lipidome uncovers early changes in adrenoleukodystrophy human cortical and spinal organoids

**DOI:** 10.1016/j.isci.2025.114339

**Published:** 2025-12-04

**Authors:** Roberto Montoro Ferrer, Yorrick R.J. Jaspers, Nicki Coveña, Nicole Breeuwsma, Inge M.E. Dijkstra, Julia Kempff, Jan-Bert van Klinken, Joke Wortel, Jan R.T. van Weering, Marc Engelen, Stephan Kemp, Vivi M. Heine

**Affiliations:** 1 Laboratory Genetic Metabolic Diseases, Department of Laboratory Medicine, Amsterdam UMC, Amsterdam Gastroenterology Endocrinology Metabolism, University of Amsterdam, Amsterdam, the Netherlands; 2 Department of Child and Adolescence Psychiatry, Emma Center for Personalized Medicine, Emma Children’s Hospital, Amsterdam UMC, Amsterdam Neuroscience, Amsterdam, the Netherlands; 3 Department of Pediatric Neurology, Emma Children’s Hospital, Amsterdam UMC, Amsterdam Leukodystrophy Center, Amsterdam Neuroscience, University of Amsterdam, Amsterdam, the Netherlands; 4 Department of Complex Trait Genetics, Centre for Neurogenomics and Cognitive Research, Amsterdam Neuroscience, Vrije Universiteit Amsterdam, Amsterdam, the Netherlands; 5 Core Facility Metabolomics, Amsterdam UMC Location University of Amsterdam, Amsterdam, the Netherlands; 6 Center for Neurogenomics and Cognitive Research, Department of Functional Genomics, Amsterdam Neuroscience, Vrije Universiteit Amsterdam, Amsterdam 1081, the Netherlands; 7 Center for Neurogenomics and Cognitive Research, Department of Clinical Genetics, Amsterdam Neuroscience, Amsterdam UMC location VUmc, Amsterdam 1081, the Netherlands

**Keywords:** Nervous system anatomy, Developmental neuroscience, Lipidomics

## Abstract

Lipids are critical for the structure, signaling, and metabolism of the central nervous system (CNS), yet their roles during human brain development remain underexplored due to limited tissue availability. X-linked adrenoleukodystrophy (ALD), a peroxisomal disorder caused by *ABCD1* mutations, disrupts very long-chain fatty acid (VLCFA) degradation, leading to axonal degeneration and demyelination. To investigate lipid dynamics in CNS development and ALD pathogenesis, we generated human induced pluripotent stem cell (hiPSC)-derived cortical and spinal cord organoids and performed lipidomics over 200 days. Lipidomic analysis revealed a dynamic lipidome, with changes in lipid abundance, saturation, and chain length reflecting neurodevelopment. ALD hiPSC-derived organoids exhibited significant lipid alterations over time, including elevated VLCFA levels and reductions in brain-relevant lipids, such as sulfatides and gangliosides, in cortical organoids. These findings provide a foundational resource for studying lipid dynamics in CNS development and emphasize the value of organoids for understanding ALD and other CNS diseases.

## Highlights

• Lipidomics shows dynamic lipid changes in human cortical and spinal organoids
• ALD organoids show early and persistent buildup of very long-chain fatty acids
• Organoid lipid changes mirror those seen in ALD patient brain tissue
• Organoids enable the study of neurodevelopmental lipid dysregulation in ALD

## Graphical abstract

**Figure:**
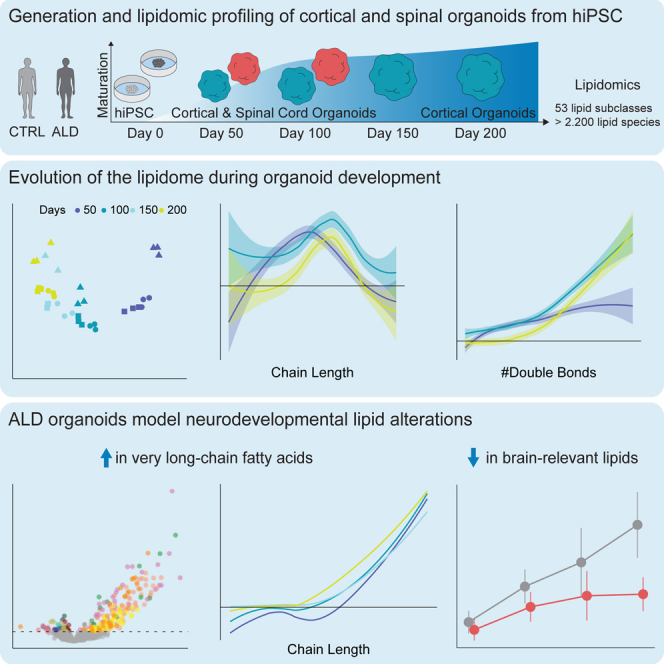

## Introduction

Lipids are a critical component of the central nervous system (CNS), playing essential roles in cell structure, signaling, and energy metabolism. However, understanding the lipid composition of the human brain during development remains a significant challenge. This is due to the limited availability of samples and the inherent difficulty in obtaining such tissue. This scarcity has impeded research into the influence of lipid changes on neurodevelopmental processes and their contribution to disorders of the CNS. To address this, three-dimensional, self-organizing structures derived from human pluripotent stem cells, known as organoids, provide new, powerful tools for modeling human CNS development and maturation *in vitro*.^1^ In addition, organoids can be regionally patterned to resemble specific CNS regions, such as the cortex and the spinal cord, to recapitulate specific characteristics associated with human CNS development. This provides an accessible and versatile approach to studying brain lipid development and its role in CNS disorders. However, despite their potential, the lipid profile of developing organoids has not yet been comprehensively studied.

Lipid dysregulation is increasingly implicated in the pathology of CNS disorders, affecting crucial processes such as neuronal signaling, synaptic function, and myelin integrity.^2^ Among these disorders, X-linked adrenoleukodystrophy (ALD) is the most common peroxisomal neurometabolic disorder (OMIM: 300100). ALD is caused by pathogenic variants in the *ABCD1* gene, which encodes a peroxisomal membrane protein responsible for the transport of very long-chain fatty acids (VLCFAs) into peroxisomes for ß-oxidation.^3^^,^^4^ From a biochemical perspective, peroxisomal dysfunction in ALD is characterized by elevated levels of VLCFA.^5^^,^^6^^,^^7^ From a clinical standpoint, the core manifestation of the disease is a slowly progressive axonal degeneration in the spinal cord and peripheral nerves, which presents in adulthood and affects both male and female patients.^8^^,^^9^^,^^10^ In addition, male patients may develop adrenocortical insufficiency and leukodystrophy (cerebral ALD), which is characterized by a progressive degeneration of the cerebral white matter.^11^ There is no simple genotype-phenotype correlation, and the unclear mechanism by which deficits in VLCFA degradation may lead to demyelination and/or axonal degeneration highlights the complexity of this disorder.^11^

To gain a deeper understanding of the lipid changes that occur in both healthy and diseased states, we generated cortical and spinal cord organoids derived from human induced pluripotent stem cells (hiPSCs) from healthy individuals and patients with ALD and performed lipidomics at multiple developmental stages, spanning up to 200 days. Lipid profiling of developing cortical and spinal organoids revealed a highly dynamic lipidome, characterized by significant changes in lipid abundance, saturation levels, and chain length throughout the development of the organoids. By mapping these lipid changes, this study provides a valuable resource for researchers to identify the most suitable *in vitro* time points for investigating specific lipids relevant to CNS disorders. Although ALD organoids followed a comparable global lipid trajectory to controls, the deficiency in VLCFA degradation resulted in a pronounced increase in VLCFA-containing species in both cortical and spinal organoids. Notably, our analysis identified a reduction in several brain-relevant lipid classes, including sulfatides and gangliosides, in ALD cortical organoids, which, to the best of our knowledge, is the first time reported in an ALD model system. Collectively, these findings emphasize the neurodevelopmental aspects of ALD and highlight the potential of CNS organoids as a model to understand ALD pathomechanisms.

## Results

### Developmental lipid trajectory profiling in organoids

**Figure 1:**
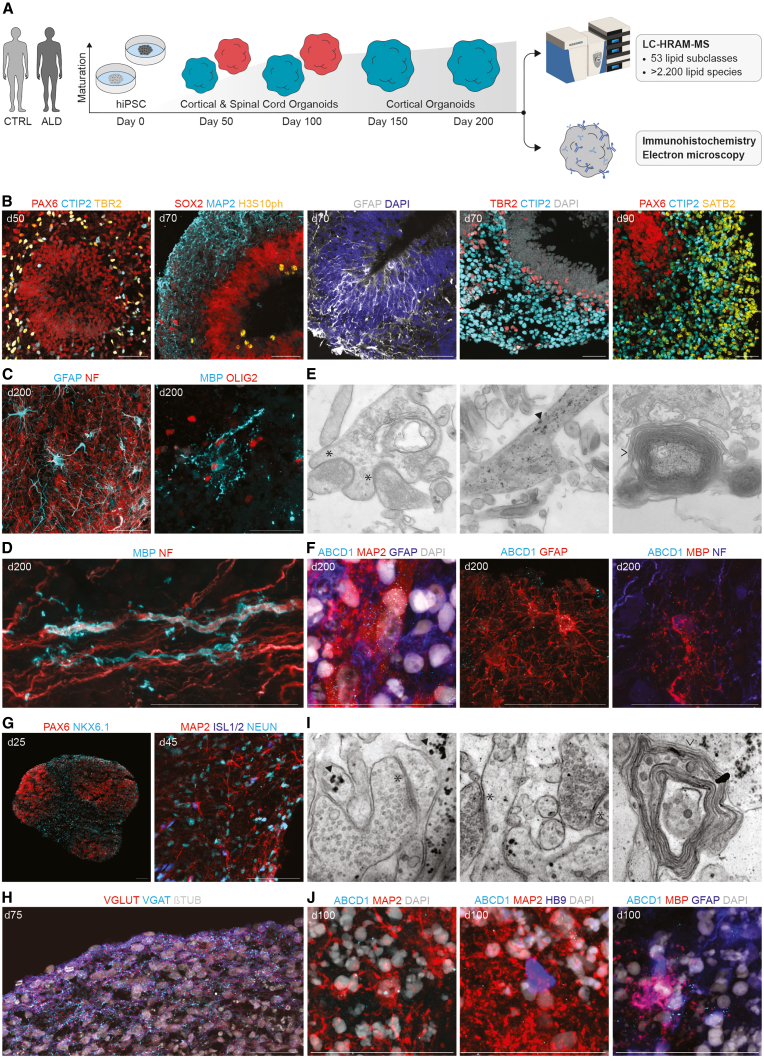
Study workflow for cortical and spinal organoids derived from hiPSCs and characterization of organoids across maturation stages (A) Schematic representation of the experimental design. Cortical and spinal cord organoids were derived from human induced pluripotent stem cells (hiPSCs) from control (CTRL) and adrenoleukodystrophy (ALD) donors. Lipidomics was performed using liquid chromatography high-resolution accurate mass spectrometry (LC-HRAM-MS). Immunohistochemistry and electron microscopy were used for structural and cellular characterization. (B) Representative confocal immunofluorescence images of control hiPSC-derived cortical organoids (hCOs). At earlier stages, hCO generated distinct populations of cycling dorsal neural progenitors (SOX2, H3S10ph, and PAX6) that organized into ventricular-like zones, with an outer subventricular-zone-like area of TBR2^+^ intermediate progenitors, and an adjacent cortical-like plate of CTIP2^+^ deep layer and SATB2^+^ upper layer neurons (scale bars, 50 μm). (C and D) At later stages, hCO are composed of GFAP^+^ astrocytes, OLIG2^+^ oligodendrocyte precursor cells, and MBP^+^ myelinating oligodendrocytes (scale bars, 50 μm). (E) Representative electron microscopy images of day 200 hCO showing mushroom-shaped dendritic spines (asterisks), glycogen particles in astrocytes (triangles), and myelin-forming oligodendrocytic processes (right pointing arrowhead). (F) Representative confocal immunofluorescence images of ABCD1 present in MAP2^+^ neurons, GFAP^+^ astrocytes, and MBP^+^ oligodendrocytes at day 200 (scale bars, 50 μm). (G and H) Representative confocal immunofluorescence images of control hiPSC-derived spinal organoids (hSO). At earlier stages, hSO generated distinct PAX6^+^ dorsal and NKX6.1^+^ ventral progenitor zones. At later stages, hSO are composed of ISL1/2^+^ motor neurons, and glutamatergic VGLUT^+^ and GABAergic VGAT^+^ synaptic vesicles (scale bars, 100 μm). (I) Representative electron microscopy images of day 100 hSO show dendritic spines (asterisk), glycogen particles in astrocytes (triangles), and myelin-forming oligodendrocytic processes (arrowhead). (J) Representative confocal immunofluorescence images of ABCD1 present in MAP2^+^ neurons, GFAP^+^ astrocytes, and MBP^+^ oligodendrocytes at day 100 hSO (scale bars, 50 μm).

To investigate the lipid profile of the human developing nervous system in ALD, we generated human cortical organoids (hCOs) and human spinal cord organoids (hSOs) from 10 patients with ALD and 3 healthy individuals following a close iteration of previously published protocols,^12^^,^^13^ with an emphasis on glia generation (Figure 1A). To profile the lipidome of developing organoids, we collected longitudinal samples of hCO at days 50, 100, 150, and 200, and of hSO at days 50 and 100. Lipidomic analysis of developing organoids using high resolution mass spectrometry resulted in the identification of 2.262 lipid species belonging to 53 lipid subclasses (Figure S1A).

The development of the human cortex *in vivo* is dependent on a meticulously orchestrated process whereby successive waves of neurons migrate along radial glial scaffolds, ultimately leading to the formation of a stratified structure comprising germinal zones and an inside-out cortical plate arrangement.^14^ To study these developmental processes *in vitro*, we characterized the organoids using immunohistochemistry and electron microscopy. Control hCO exhibited an organization that closely mirrors *in vivo* dorsal cortical development. At earlier stages (day 50 to day 70), hCO mainly contained dense PAX6^+^ and SOX2^+^ neural progenitor populations lining an internal lumen, reminiscent of a ventricular zone (Figure 1B). Actively dividing H3S10PH^+^ cells are located at the apical surface, indicating ongoing progenitor cell division. Moreover, TBR2^+^ intermediate progenitors lined up adjacent to the ventricular zone, resembling a subventricular zone. At later stages (day 90), next to the germinal zone, we observed a distinct cortical-like plate composed of CTIP2^+^ deep-layer and SATB2^+^ upper-layer cortical neurons, showing a rough spatial separation. At day 200, hCO were also composed of mature GFAP^+^ astrocytes and MBP^+^ oligodendrocytes with processes wrapping axons (Figures 1C and 1D). In addition, we confirmed with electron microscopy the presence of synapses, multiple layers of myelin wrapping axons, and astrocytes rich in glycogen-granules at day 200 (Figure 1E). We next assessed the presence of ABCD1 in control hCO. At day 50, ABCD1 was present throughout the tissue, including densely packed neural progenitor areas, as well as in young CTIP2^+^ deep-layer neurons (Figure S1B). At day 200, we observed ABCD1 present in many GFAP^+^ astrocytes, and in some MAP2^+^ neurons and MBP^+^ oligodendrocytes (Figures 1F and S1C–S1E).

The development of the spinal cord *in vivo* requires the precise patterning of dorsal and ventral domains driven by morphogen gradients, leading to the specification of progenitor zones and the differentiation of motor neurons and interneurons.^15^ To model these processes *in vitro*, we characterized developing control hSO and observed the formation of both dorsal and ventral regions, defined by PAX6^+^ dorsal domains and NKX2.1^+^ ventral domains, indicative of proper regional patterning (Figure 1G). We confirmed the presence of ISL1/2^+^ and NeuN^+^ motor neurons at day 45. We also observed the presence of excitatory VGLUT^+^ and inhibitory VGAT^+^ synaptic vesicle markers at day 75 (Figure 1H). By using electron microscopy, we observed the presence of synapses, myelin wrapped axons, and astrocytes containing glycogen granules at day 100 (Figure 1I). At day 100, we observed ABCD1 presence in MAP2^+^ neurons, GFAP^+^ astrocytes, and MBP^+^ oligodendrocytes, but not in HB9^+^ motor neurons (Figures 1J and S1F–S1H). These findings collectively demonstrate that hCO and hSO recapitulate key developmental milestones of the human cortex and spinal cord *in vitro*, providing a robust model to investigate the lipid dynamics in healthy and diseased states.

### Lipidomic profiling of cortical organoid development

**Figure 2:**
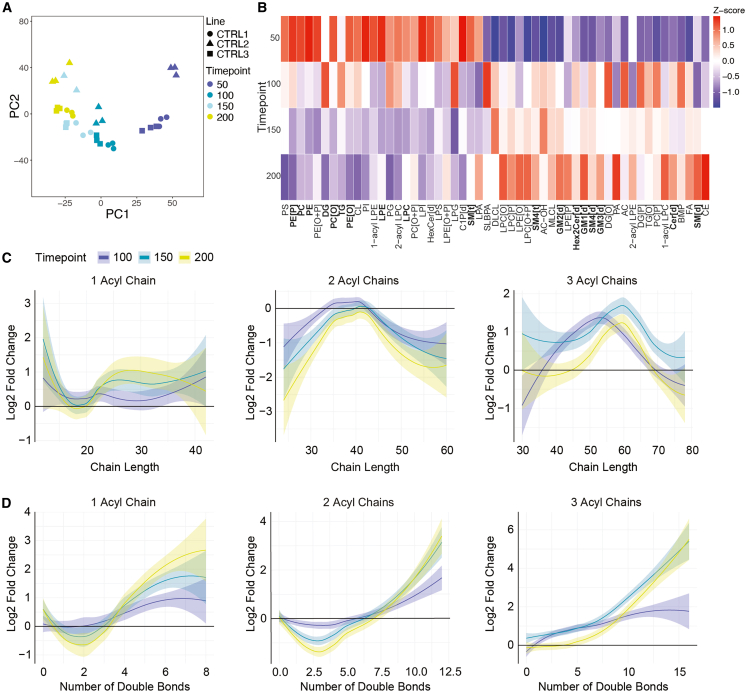
Characterization of cortical organoids and lipidomic profiling across maturation stages (A) Principal component analysis (PCA) of lipidomic profiles shows distinct clustering of cortical organoids by maturation timepoint (color-coded) and individual lines (shapes). *n* = 3 lines, 4 timepoints, 3 samples per line. (B) Heatmap shows the Z-scores of lipid class abundance across four timepoints (days 50, 100, 150, and 200). Each lipid class displays dynamic changes in abundance during organoid maturation. (C) Log2 fold changes of lipid classes with 1, 2, and 3 acyl chains as a function of total acyl chain length across timepoints (days 100, 150, 200, relative to day 50), fitted with LOESS smoothing. (D) Log2 fold changes of lipid classes with 1, 2, and 3 acyl chains as a function of the total number of double bonds across timepoints (days 100, 150, 200, relative to day 50), fitted with LOESS smoothing.

Principal component analysis (PCA) of the control hCO lipidome (Figure 2A) revealed a distinct clustering of samples driven primarily by differentiation time, underscoring a dynamic lipidome that evolves over time. This temporal evolution was also reflected at the level of lipid classes (Figure 2B). At day 50, elevated levels of phosphatidylcholine (PC), phosphatidylethanolamine (PE) and their ether-linked forms (PC[O], PE[O], PE[P]) were observed, along with triacylglycerols (TGs) and diacylglycerols (DGs). At day 100, increased levels of lysophospholipids, including lysophosphatidylcholine (LPC) and lysophosphatidylethanolamine (LPE), as well as sphingomyelin (SM[d], SM[t]) and ceramides (Cer[d]) were observed. These trends continued at day 150, with the further enrichment of sphingomyelin and ceramides, along with gangliosides (GM1[d], GM3[d]) and sulfatides (SM4[d]), accompanied by a reduction in TG and DG levels. By day 200, lipid profiles stabilized, characterized by sustained levels of sphingomyelin, ceramides, hexosylceramides (Hex2Cer[d]), and gangliosides.

To further investigate the evolving lipid profile, we analyzed alterations in lipid chain length and degree of unsaturation in hCO in comparison to day 50 (Figures 2C and S2). Single-acyl chain-containing lipids with shorter acyl chains (<20 carbons) decreased over time, while medium to long acyl chains (>20 carbons) increased, peaking at day 200. Two-acyl chain-containing lipids (∼30–50 carbons) initially decreased at days 100 and 150 but were increased by day 200, while three-acyl chain-containing lipids (∼40–70 carbons) showed a continued increase, especially at day 200. Across all lipid groups, highly polyunsaturated species (≥6 double bonds) progressively increased (Figures 2D and S3). Overall, these shifts reflect the transition from simpler lipids to longer, highly unsaturated species as the organoids develop.

### Lipidomic profiling of spinal organoid development

**Figure 3:**
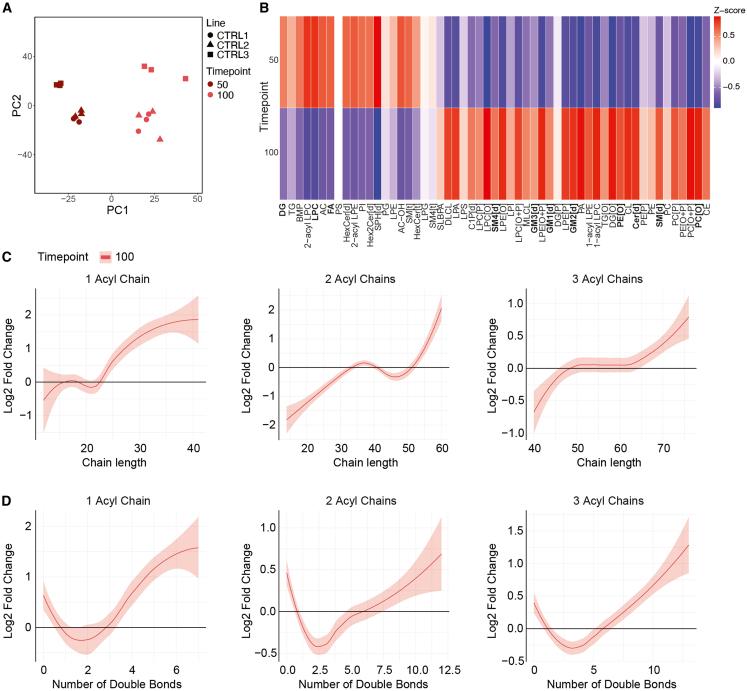
Characterization of spinal cord organoids and lipidomic profiling across maturation stages (A) Principal component analysis (PCA) of lipidomic profiles shows the distinct clustering of spinal cord organoids by maturation timepoint (color-coded) and individual lines (shapes). *n* = 3 lines, 2 timepoints, 3 samples per line. (B) Heatmap shows the Z-scores of lipid class abundance across four timepoints (days 50 and 100). Each lipid class displays dynamic changes in abundance during organoid maturation. (C) Log2 fold changes of lipid classes with 1, 2, and 3 acyl chains as a function of total acyl chain length at day 100, relative to day 50, fitted with LOESS smoothing. (D) Log2 fold changes of lipid classes with 1, 2, and 3 acyl chains as a function of the total number of double bonds at day 100 relative to day 50, fitted with LOESS smoothing.

PCA of the control hSO lipidomes revealed a clear separation between differentiation days 50 and 100 (Figure 3A). At the lipid class level, hSO exhibited developmental lipid shifts similar to those observed in hCO organoids, with distinct changes in composition between days 50 and 100 (Figure 3B). At day 50, DG, LPC, and free fatty acids (FAs) were relatively more abundant. By day 100, the lipid profile shifted, showing an increased abundance of more complex sphingolipids, including Cer, SM[d], SM[t], and SM4[d], gangliosides GM1[d], GM2[d], and GM3[d], and ether-linked phospholipids (PC[O] and PE[O]).

An analysis of total lipid chain length, including lipids with one, two, or three fatty acids, showed a progressive increase in chain length over time (Figures 3C and S4). In addition, trends in lipid saturation showed an increase in PUFA-containing lipid chains in hSO by day 100 (Figures 3D and S5). At day 100, hSO showed an enrichment of longer chain and more unsaturated lipids compared to day 50. Single acyl chain-containing lipids with >25 carbons and ≥3 double bonds were increased. The two-acyl chain-containing lipids with very long chains (>50 carbons) and high levels of unsaturation (>6 double bonds) also increased, indicating the synthesis of complex phospholipids. Similarly, three-acyl chain-containing lipids with longer chains with >70 carbons and >10 double bonds were increased. These changes reflect a shift toward complex lipids that are critical for neuronal development.

### Adrenoleukodystrophy results in very long-chain fatty acid incorporation in complex lipids across organoid differentiation

**Figure 4:**
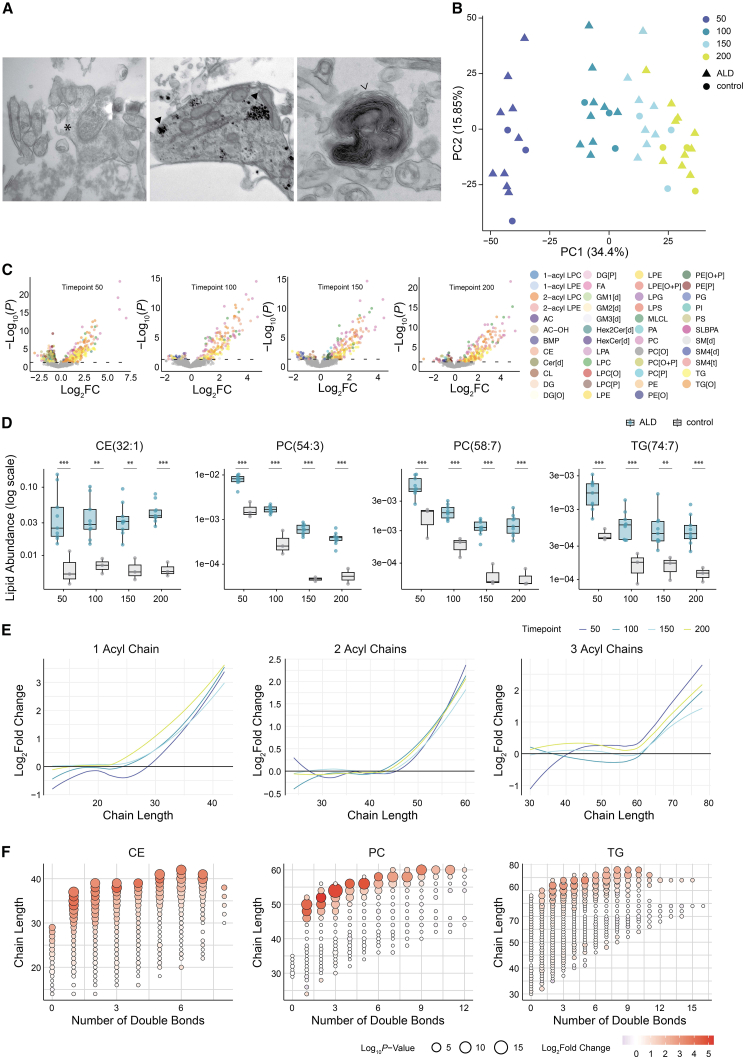
Lipidomic profiling reveals changes in lipid composition in ALD and control cortical organoids across maturation stages (A) Representative electron microscopy images of day 200 ALD hCO show dendritic spines (asterisks), glycogen particles in astrocytes (triangles), and myelin-forming oligodendrocytic processes (right pointing arrowhead). (B) Principal component analysis (PCA) of lipidomic profiles of cortical organoids across maturation stages (days 50, 100, 150, and 200) comparing ALD (triangles) and control (circles) samples. Points are color-coded by timepoint, illustrating clear clustering patterns based on maturation and disease status. Data represent median lipid profiles of 3 samples per line. *n* = 12 lines (9 ALD lines, 3 control), 4 timepoints. (C) Volcano plots illustrate Log2 fold change (Log2 FC) versus -Log10 (*p*-value) for lipid species at each timepoint (days 50, 100, 150, and 200) comparing ALD to controls. Each point represents one lipid species, color coded by lipid subclass. Statistical significance was determined using a linear mixed-effects model. (D) Boxplots show the abundance (log scale) of representative lipid species (CE(32:1), PC(54:3), PC(58:7), TG(74:7)) across timepoints for ALD (blue) and control (gray) organoids. Statistical significance for comparisons between ALD and control is indicated above the boxes at each time point. Statistical significance was determined using a linear mixed-effects model. Data represent median lipid profiles of 3 samples per line. *n* = 12 lines (9 ALD lines, 3 control), 4 timepoints. Boxes show the interquartile range (IQR) with the median; whiskers extend to 1.5 × IQR. ∗∗ FDR ≤0.01, ∗∗∗ FDR ≤0.001. FDR values for CE(32:1) were 8.95×10^−4^ (T50), 3.03×10^−3^ (T100), 5.15×10^−3^ (T150), and 1.21×10^−4^ (T200); for PC(54:3) 1.94×10^−9^ (T50), 2.04×10^−8^ (T100), 4.63×10^−12^ (T150), and 6.22×10^−19^ (T200); for PC(58:7) 2.09×10^−5^ (T50), 2.72×10^−5^ (T100), 3.56×10^−9^ (T150), and 5.28×10^−9^ (T200); and for TG(74:7) 4.62×10^−4^ (T50), 6.01×10^−4^ (T100), 5.93×10^−3^ (T150), and 3.16×10^−4^ (T200). (E) Line plots show Log2 fold change for lipid species with 1, 2, and 3 acyl chains as a function of acyl chain length, comparing ALD to controls. Trends are shown for days 50, 100, 150, and 200, fitted with LOESS smoothing. (F) Bubble plots illustrate the relationship between acyl chain length and the number of double bonds for cholesteryl esters (CEs), phosphatidylcholines (PCs), and triglycerides (TGs) at day 200, comparing ALD to controls. Bubble size corresponds to -Log10 (*p*-value) and color intensity indicates Log2 fold change. Statistical significance was determined using a linear mixed-effects model.

We generated hiPSC from primary skin fibroblasts of 10 male patients with ALD and characterized them for pluripotency (Figure S6). Immunostaining analysis of neuronal differentiation, cell proliferation, and cell death showed no differences between ALD and control organoids (Figure S7). At day 200, we confirmed with electron microscopy the presence of synapses, astrocytes, and myelin-forming oligodendrocytes in ALD hCO (Figure 4A). PCA of lipidomics revealed that the ALD hCO clustered closely with differentiation day-matched controls (Figure 4B). Furthermore, the lipid classes in ALD hCO followed a similar evolutionary trajectory to control hCO, also showing comparable trends in chain length and saturation (Figures S8 and S9). However, significant differences in lipid levels were observed between ALD and control samples at all time points. The most pronounced changes were elevations in CE, PC, and TG lipid species (Figure 4C). Examples of elevated lipids in ALD are shown in Figure 4D. The elevated single acyl chain-containing lipids in ALD, such as CE and LPC, exhibited acyl chain lengths greater than 26 carbons and up to 8 double bonds (Figures 4D, 4E, and S10). The elevated two-acyl chain-containing lipids, such as PC and DG, exhibited total chain lengths greater than 46 carbons with up to 12 double bonds. The elevated three-acyl chain-containing lipids, such as TG, exhibited chain lengths greater than 62 carbons, with up to 16 double bonds. Taken together, this indicates that the majority of elevated lipids in ALD are VLCFA-containing lipid species. Interestingly, the fold change between control and ALD hCO samples increased with the total fatty acyl chain length of the lipid species, irrespective of the number of double bonds (Figures 4F and S11).

**Figure 5:**
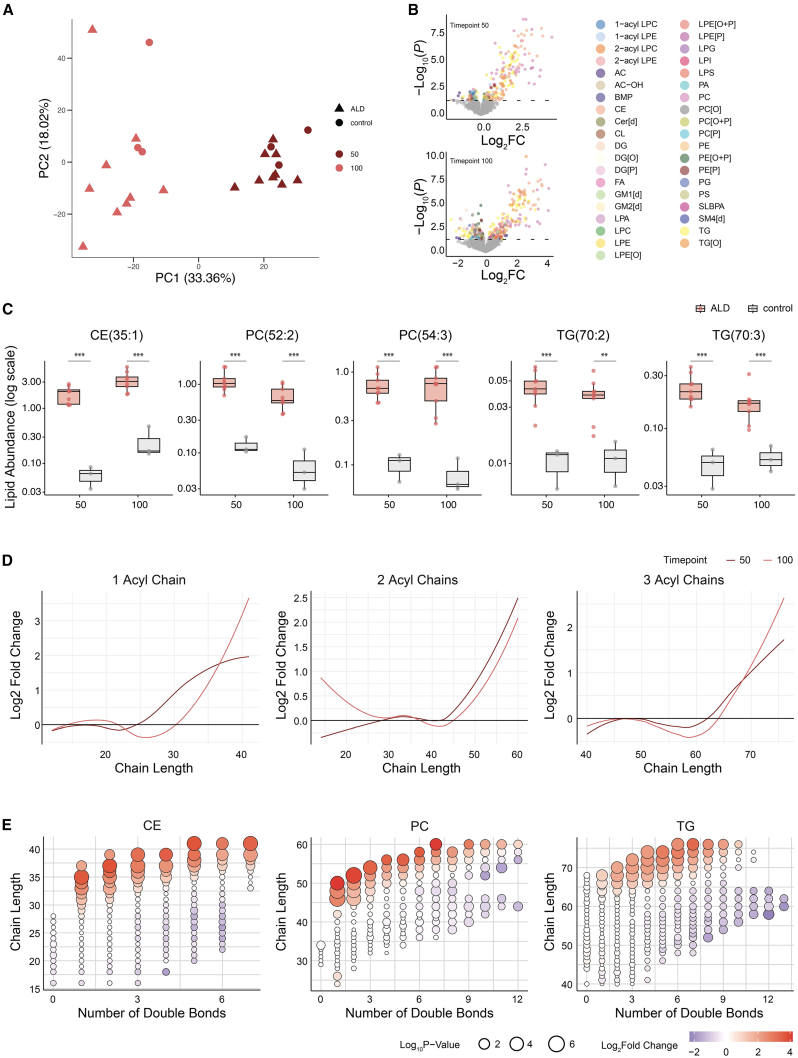
Lipidomic profiling reveals changes in lipid composition in ALD and control spinal cord organoids across maturation stages (A) Principal component analysis (PCA) of lipidomic profiles of spinal cord organoids across maturation stages (days 50 and 100), comparing ALD (triangles) and control (circles) samples. Points are color-coded by timepoint, illustrating clear clustering patterns based on maturation and disease status. Data represent median lipid profiles of 3 samples per line (*n* = 2 timepoints, 9 ALD lines, 3 control lines). (B) Volcano plots illustrate Log2 fold change (Log2 FC) versus -Log10 (*p*-value) for lipid species at each timepoint (days 50 and 100), comparing ALD to controls. Each point represents a lipid species, color-coded by lipid subclass. Statistical significance was determined using a linear mixed-effects model. (C) Boxplots show the abundance (log scale) of representative lipid species (CE(35:1), PC(52:2), PC(54:3), TG(70:2), and TG(70:3)) across timepoints for ALD (red) and control (gray) organoids. Statistical significance for comparisons between ALD and control is indicated above the boxes at each time point. Statistical significance was determined using a linear mixed-effects model. Data represent median lipid profiles of 3 samples per line. *n* = 12 lines (9 ALD lines, 3 control). Boxes show the interquartile range (IQR) with the median; whiskers extend to 1.5 × IQR. ∗∗ FDR ≤0.01, ∗∗∗ FDR ≤0.001. FDR values for CE(35:1) were 4.58×10^−6^ (T50) and 9.19×10^−6^ (T100); for PC(52:2) 4.58×10^−6^ (T50) and 5.84×10^−7^ (T100); for PC(54:3) 1.40×10^−4^ (T50) and 4.10×10^−5^ (T100); for TG(70:2) 3.24×10^−4^ (T50) and 1.30×10^−3^ (T100); and for TG(70:3) 1.16×10^−4^ (T50) and 2.05×10^−4^ (T100). (D) Line plots show Log2 fold change for lipid species with 1, 2, and 3 acyl chains as a function of acyl chain length, comparing ALD to controls. Trends are shown for days 50 and 100, fitted with LOESS smoothing. (E) Bubble plots illustrate the relationship between acyl chain length and the number of double bonds for cholesteryl esters (CEs), phosphatidylcholines (PCs), and triglycerides (TGs) at day 100, comparing ALD to controls. Bubble size corresponds to -Log10 (*p*-value) and color intensity indicates Log2 fold change. Statistical significance was determined using a linear mixed-effects model.

Similar to hCO, PCA analysis showed that ALD hSO samples clustered with differentiation day-matched controls (Figure 5A), demonstrating that ALD hSO followed a comparable developmental trajectory to control hSO (Figures S12 and S13). Also here, ALD hSO exhibited significant increases in CE, PC, and TG lipids at both time points (Figures 5B and 5C). Examples of elevated lipids in ALD hSO are shown in Figure 5C. Elevated lipids in ALD hSO were mainly VLCFA-containing species (Figures 5C–5E and S14). And, also consistent with findings in hCO, the fold change between control and ALD hSO increased with the total fatty acyl chain length of the lipid species, regardless of the number of double bonds (Figures 5D, 5E, and S15).

Taken together, these results demonstrate that the lipid profiles of developing ALD hCO and hSO follow maturation patterns similar to those of healthy control organoids. However, the underlying biochemical deficiency in ALD results in the incorporation of VLCFA into complex lipids, a feature consistently observed in both cortical and spinal organoids at all time points.

### Brain-relevant lipids are decreased in adrenoleukodystrophy human induced pluripotent stem cell-derived cortical organoids

**Figure 6:**
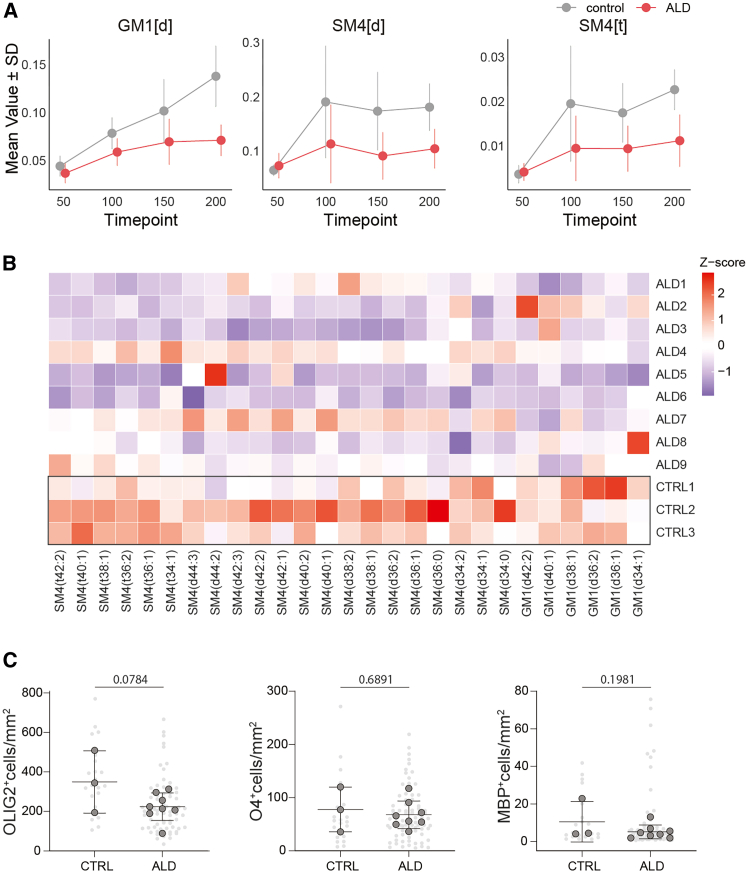
Altered lipid metabolism in ALD cortical organoids (A) Line plots show the mean values ±SD for GM1[d] and SM4[d] across maturation timepoints (days 50, 100, 150, 200) in control (gray) and ALD (red) organoids. FDR values of the overall group difference across all timepoints for GM1[d] were 0.126 (*p* = 0.0167); for SM4[d] 0.0854 (*p* = 0.00444); and for SM4[t] 0.126 (*p* = 0.0168). Data represent median lipid profiles of 3 samples per line. *n* = 12 lines (9 ALD lines, 3 control), 4 timepoints. Statistical significance was determined using a linear mixed-effects model. (B) Heatmap of Z-scores for individual SM4 and GM1 lipid species across maturation stages in control and ALD hCO. Each row represents a lipid species, and each column represents a hiPSC line. Lipid species show distinct profiles between the ALD and control groups. Data represent median lipid profiles of 3 samples per line. *n* = 12 lines (9 ALD lines, 3 control), 4 timepoints. (C) Quantification of OLIG2^+^, O4^+^, and MBP^+^ cells at day 200 hCO. Quantification was performed in 3 whole tissue sections from 3 to 4 organoids per hiPSC line. *p* values were calculated using two-tailed nested *t* test. Data are represented as mean ± SD.

Next, we analyzed the trends in lipid classes in hCO, focusing on those classes that displayed a consistent lipid trajectory and showed differences by day 200. We found a trend of reduced levels of gangliosides (GM1[d]) and sulfatides (SM4[d] and SM4[t]) in ALD hCO (Figure 6A). Moreover, the majority of the lipid species identified within these lipid classes showed a reduction compared to controls (Figure 6B). Given that ceramides are a common precursor for gangliosides and sulfatides, we analyzed their associated pathways and observed only minor changes in a few lipid classes at day 50 (Figure S16).

Because sulfatides are abundant in myelin and, together with gangliosides, are essential for myelin maintenance,^16^^,^^17^ we assessed the oligodendrocyte lineage at day 200 ALD hCO. We quantified the oligodendrocyte progenitor cell (OPC) population with the marker OLIG2, pre-oligodendrocyte cells with O4, and mature oligodendrocytes with MBP (Figure 6C). We observed a trend toward a reduction of the OPC population in ALD hCO, but no significant changes were observed in pre-oligodendrocytes or oligodendrocytes.

These findings suggest a potential impairment in the lipid metabolism of gangliosides and sulfatides critical for myelin maintenance in ALD hCO, while the overall population of oligodendrocyte lineage remains stable.

### Increased phosphatidylethanolamine plasmalogen degradation in adrenoleukodystrophy human induced pluripotent stem cell-derived cortical organoids

**Figure 7:**
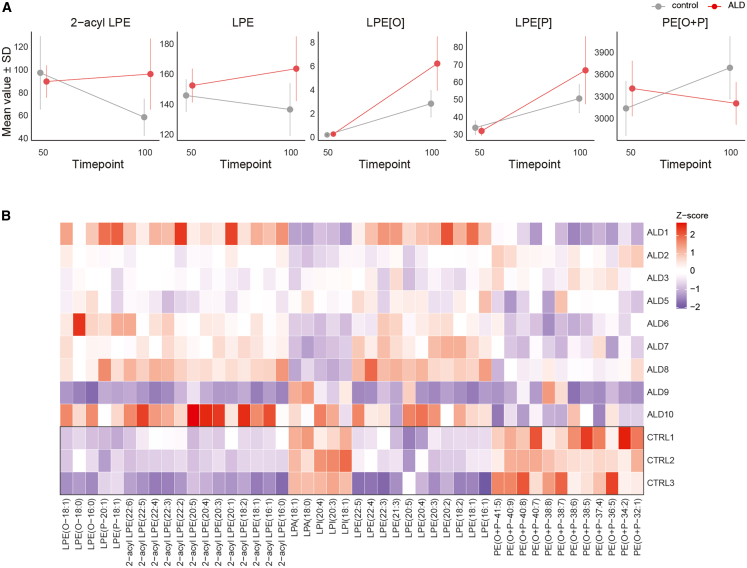
Altered lipid metabolism in ALD spinal organoids (A) Line plots show mean values ±SD for lipid classes across two timepoints (days 50 and 100) in control (gray) and ALD (red) organoids. Lipid subclasses include 2-acyl-LPE, LPE, LPE[O], LPE[P], and PE[O + P]. FDR values of the overall group difference across all timepoints for 2-acyl LPE were 0.117 (*p* = 0.00675); for LPE 0.120 (*p* = 0.0152); for LPE[P] 0.193 (*p* = 0.0385); for LPE[O] 0.117 (*p* = 0.0124); and for PE[O + P] 0.0988 (*p* = 0.00359). Data represent median lipid profiles of 3 samples per line. *n* = 12 lines (9 ALD lines, 3 control), 2 timepoints. Statistical significance was determined using a linear mixed-effects model. (B) Heatmap of Z-scores for individual lipid species within lipid classes across samples from ALD and control hSO. Each row represents a lipid species, and each column represents an hiPSC line. ALD organoids show distinct lipidomic profiles compared to controls. Data represent median lipid profiles of 3 samples per line. *n* = 12 lines (9 ALD lines, 3 control), 2 timepoints.

We extended the lipid class trend analysis to hSO, focusing on lipid classes that showed significant differences at day 100. The analysis revealed a reduction in the levels of plasmalogen PE[O + P], LPA, and LPI in ALD hSO compared to controls (Figure 7A). Conversely, the levels of LPE, LPE[O], LPE[P], and 2-acyl-LPE were increased. Notably, the majority of lipid species identified within these lipid classes showed a reduction compared to controls (Figure 7B). Taken together, these findings suggest a potential increase in the degradation of PE plasmalogen in ALD hSO.

## Discussion

This study presents a comprehensive lipidomic analysis of hiPSC-derived cortical organoids (hCOs) and spinal cord organoids (hSOs) derived from both healthy individuals and patients with ALD. By leveraging CNS organoids as model systems, our work provides critical insights into the lipid landscape of the developing human nervous system.

hCO and hSO showed an evolution of their lipid profile over time, likely reflecting the metabolic demands associated with CNS development. High levels of structural lipids (PC and 2PE) and energy storage lipids (TG and DG) at early timepoints arise from the demands associated with rapid neuronal proliferation and membrane biogenesis. Both hCO and hSO showed increasing levels of sphingomyelin, ceramides, gangliosides, and sulfatides over time, with gangliosides contributing to neuronal signaling and synaptic function and sulfatides, along with sphingomyelins, being essential for myelin formation and maintenance.^2^^,^^18^^,^^19^ At the latest time point, the abundance of these lipid classes stabilizes, reflecting the maturation of the organoids. Across all lipid classes, polyunsaturated species progressively increased in hCO and hSO over time. These lipids are critical for membrane function and fluidity, and support synaptic plasticity and neuronal polarity by modulating membrane dynamics and signaling.^2^^,^^20^^,^^21^ While vascularization is absent in our model, structural evidence, including synaptic and myelin-wrapping features and mature astrocyte morphology at day 200, suggests continued viability beyond 100 days, supporting the utility of these organoids for studying long-term developmental lipid dynamics and disease mechanisms.^22^ Collectively, the trajectory of these lipids reflects the progressive lipid requirement for architectural complexity and neuronal specialization during CNS development. This study provides a foundational lipid profile of developing organoids, serving as a reference point for future research. We anticipate that incorporating additional developmental time points and alternative organoid models will refine and expand our understanding of lipid dynamics in both healthy and pathological states.

The lipid profiles of hCO and hSO derived from patients with ALD demonstrate maturation trajectories comparable to those of healthy controls. However, ALD hCO and hSO display aberrant accumulation of VLCFA in a number of lipid classes at all timepoints. This aligns with the known biochemical hallmark of ALD, namely the accumulation of VLCFA such as C24:0 and C26:0.^23^^,^^24^ Moreover, the most pronounced VLCFA accumulation was observed in PC, TG, and CE lipid classes. This finding is in line with previous lipidomics analyses of ALD patient samples.^9^^,^^25^^,^^26^^,^^27^^,^^28^^,^^29^^,^^30^ Plasma studies showed that VLCFA accumulation occurs across multiple lipid classes but is most pronounced in LPC, PC, and TG.^9^^,^^29^ Similarly, analyses of ALD fibroblasts and astrocytes revealed that VLCFA primarily accumulate in CE, PC, and TG.^28^^,^^30^^,^^31^ Importantly, postmortem brain tissue from patients with ALD also displayed elevated VLCFA levels in PC and CE.^25^^,^^26^^,^^27^ Together, these observations demonstrate that the lipid alterations detected in organoids parallel VLCFA signatures observed across different patient-derived materials.

The incorporation of VLCFA into phosphatidylcholine lipids is presumed to disrupt the structure and function of cellular membranes, affecting membrane fluidity, permeability, and cellular signaling.^32^^,^^33^ In particular, excessive incorporation of VLCFA into myelin-associated PC may compromise the integrity of myelin membranes. Similarly, it is possible that the incorporation of VLCFA into TG and CE could impair their function by disrupting lipid metabolism, lipid droplet formation, and degradation.^34^ The accumulation of VLCFA in TG and CE may indicate a protective response whereby VLCFA is sequestered in lipid droplets to mitigate the lipotoxic effects of VLCFA.

Compared to control, both GM1 and sulfatides (SM4[d], SM4[t]) are reduced in ALD hCO. Altered levels of gangliosides and sulfatides have also been reported in the white matter of patients with ALD.^35^^,^^36^ Specifically, GM1 was reduced, while the minor gangliosides GM2 and GM3 were increased. To our knowledge, this is the first demonstration of a reduction in ganglioside and sulfatide levels in an ALD model system, highlighting that patient-derived organoids can reproduce lipid features observed *in vivo*.

The reduction of sulfatides observed in the white matter of ALD brains is consistent with the loss of myelin, as sulfatides are integral myelin lipids and, together with galactosylceramides, comprise nearly one-third of myelin lipids.^2^^,^^37^ In ALD hCO, we observed a marked reduction in sulfatides and no differences in the number of MBP^+^ oligodendrocyte or O4^+^ pre-oligodendrocyte cells,^38^ suggesting that the observed reduction in sulfatides occurs despite a stable population of oligodendrocyte lineage cells. While sulfatides are not essential for myelin formation, they are critical for myelin maintenance.^16^ Similarly, gangliosides are critical for maintaining myelin integrity because they cooperate with sulfatides to stabilize axo-glial junction proteins such as MAG and NF155, which are critical for maintaining the integrity of myelinated nerves.^17^ These findings suggest that while the myelinating cell population is intact, lipids critical for myelin integrity are compromised. The recapitulation of these changes in hCO suggests that the reduction of sulfatides and gangliosides may play a role in the pathogenesis of ALD. Collectively, these findings highlight the potential of hCO to recapitulate specific lipid dysregulations observed in human ALD pathology.

In ALD hSO, we found decreased levels of PE[O + P] and increased levels of its lysophospholipid derivatives, including LPE, LPE[O], LPE[P], and 2-acyl-LPE. The observed increase in lysophospholipid derivatives in ALD hSO suggests an enhanced degradation of PE by phospholipase A2. A reduction in ether-linked PE has been previously reported in brain tissue from patients with ALD and in brain tissue from the *Abcd1* null mouse model.^39^ This reduction was associated with increased oxidative stress, as evidenced by increased reactive aldehydes and oxidized proteins.^39^ Notably, similar patterns of plasmalogen reduction have been identified in brain tissue from patients with Alzheimer’s disease, where reduced PE plasmalogen levels were associated with increased VLCFA levels and neurofibrillary tangles.^40^ Increased oxidative stress has also been associated with plasmalogen reduction in Alzheimer’s disease,^41^ suggesting a potential role for oxidative damage in neurodegenerative disorders. Taken together, the altered ether-linked PE profile observed in ALD hSO may serve as an early indicator of ALD pathology.

In conclusion, this study demonstrates the potential of hiPSC-derived organoids as a powerful model for elucidating the mechanisms underlying lipid dysregulation associated with ALD. By identifying key alterations in lipid profiles, including VLCFA accumulation, a reduction in key myelin-relevant lipids such as sulfatides and gangliosides in hCO, and a decrease in ether-linked PE in hSO, we provide new insights into the early pathophysiology of ALD. These findings provide evidence for an early neurodevelopmental disruption in ALD, and ultimately, this work paves the way for the development of more effective therapeutic strategies.

### Limitations of the study

This study provides a descriptive characterization of lipid changes during human neurodevelopment and disruption of these changes in ALD. We acknowledge that, as a model system, organoids predominantly recapitulate the early stages of brain development and do not fully capture the cellular diversity, structural organization, or metabolic complexity of the mature human brain. Nevertheless, ALD organoids reproduce essential biochemical features of ALD, such as the accumulation of VLCFAs in complex lipids, providing an opportunity to study disease-relevant lipid alterations in human neurons and glia. Integrating functional assays in future efforts will help elucidate the cellular and molecular mechanisms underlying ALD pathogenesis.

## Resource availability

### Lead contact

Further information and requests for resources and reagents should be directed to and will be fulfilled by the lead contact, Vivi M. Heine (vm.heine@amsterdamumc.nl).

### Materials availability

This study did not generate new materials or reagents.

### Data and code availability

The Lipidomics data have been deposited in the MetaboLights repository with the study identifier MTBLS12973.^42^ This study did not generate any codes. Additional information is available from the lead contact upon request.

## Acknowledgments

Details of funding: This study was financially supported by grants from the 10.13039/501100008731European Leukodystrophy Association (ELA International), grant number: 2019-020C2 (to SK and VH), Stichting Metakids, grant number: 2023-102 (to SK and VH), the Netherlands Organization for Scientific Research (NWO) (VIDI 016.196.310) (to ME), and Amsterdam UMC (FlexOIO) (to ME).

## Author contributions

R.M.F., M.E., S.K., and V.H. conceived the project. M.E., S.K., and V.H. obtained funding. R.M.F. performed the organoid experiments. Y.J. performed the lipidomics experiments. R.M.F., Y.J., N.C., N.B., I.D., and J.K. performed the laboratory analyses. R.M.F., Y.J., N.C., and J.B.K. analyzed the data. J.W. and Jv.W. performed the electron microscopy. S.K. and V.H. provided the resources for the project. M.E., S.K., and V.H. supervised the project. R.M.F., Y.J., M.E., S.K., and V.H. wrote the article.

## Declaration of interests

R.M.F., Y.J., N.C., N.B., I.D., J.K., J.B.K., J.K., Jv.W., and V.H. declare that they have no conflict of interest. M.E. participates in the advisory board of the United Leukodystrophy Foundation (unpaid); S.K. participates in advisory boards for ALD Connect (unpaid), the European Leukodystrophy Association (unpaid), Alex, The Leukodystrophy Charity (unpaid), and the United Leukodystrophy Foundation (unpaid).

## STAR★Methods

### Key resources table

**Table:** 

REAGENT or RESOURCE	SOURCE	IDENTIFIER
**Chemicals**		

Lipidomics	MetaboLights	MTBLS12973

**Antibodies**		

PAX6	DSHB	AB_528427
CTIP2	ABCAM	AB18465
TBR2	ABCAM	AB23345
SOX2	MERCK	AB5603
MAP2	MERCK	AB5543
H3S10ph/PH3	Cell signaling	#9706
GFAP	MERCK	#G3893
SATB2	ABCAM	AB51502
NF	MERCK	N4142
MBP	ABCAM	AB7349
OLIG2	MERCK	AB9610
ABCD1	ABCAM	AB197013
NKX6.1	DSHB	#F55A10
ISL1/2	ABCAM	#AB109517
NEUN	MERCK	MAB377
VGLUT	Synaptic Systems	#135 402
VGAT	Synaptic Systems	#131 004
HB9	DSHB	#81.5C10-s
NESTIN	BD Bioscience	#611658
O4	MERCK	MAB345
CC3	Cell Signaling	#9661
Secondary antibodies 488 Goat anti Rabbit	Thermo Scientific	# A-11008
Secondary antibodies 488 Goat anti Mouse	Thermo Scientific	# A-11001
Secondary antibodies 488 Goat anti Rat	Thermo Scientific	# A-11006
Secondary antibodies 594 Goat anti Rabbit	Thermo Scientific	# A-11012
Secondary antibodies 594 Goat anti Mouse	Thermo Scientific	# A-11005
Secondary antibodies 594 Goat anti Rat	Thermo Scientific	# A-11007
Secondary antibodies 647 Goat anti Guinea Pig	Thermo Scientific	# A-21450
Secondary antibodies 647 Goat anti Chicken	Thermo Scientific	# A-21449

**Chemicals, peptides, and recombinant proteins**		

Vitronectin XF	Stem Cell Technologies	#07180
Gentle Cell Dissociation Reagent	Stem Cell Technologies	#100-0485
TeSR™-E8™ medium	Stem Cell Technologies	#05990
Accutase	MERCK	#SF006
Ultra-low attachment U-bottom 96-well plates	Corning	#CLS7007
ROCK inhibitor	Selleckchem	#S1049
FGF2	Peprotech	#AF-100-18B
dorsomorphin	Selleckchem	#S7306
SB431542	Selleckchem	#S1067
DMEM/F12	Thermo Fisher Scientific	#11530566
Neurobasal Medium	Thermo Fisher Scientific	#11570556
N2	Thermo Fisher Scientific	#15410294
B27-A	Thermo Fisher Scientific	#15440584
human insulin	MERCK	#I9278
L-glutamine	Thermo Fisher Scientific	#11500626
non-essential amino acids	Thermo Fisher Scientific	#11140035
β-mercaptoethanol	Thermo Fisher Scientific	#21985023
penicillin-streptomycin	Thermo Fisher Scientific	#15140122
EGF	Peprotech	#AF-10015
Poly(2-hydroxyethyl methacrylate)-treated	MERCK	#P3932
24-well plates	VWR	#391-3370
NT3	Peprotech	#450-03
BDNF	Peprotech	#450-02
cAMP	MERCK	#D0260
Ascorbic acid	MERCK	#A4544
PDGF	Bio-techne	#221-AA
IGF1	Peprotech	#100-11
T3	MERCK	#T6397
CHIR 99021	Cayman Chemicals	#13122
B27	Thermo Fisher Scientific	#15360284
RA	ReproCell	#04-0021
SAG	Cayman Chemicals	#11914
PFA	Electron Microscopy Sciences	#15710
OCT	Tissue-Tec Oct Compound	#4583
goat serum	Thermo Scientific	#16210
BSA	MERCK	#A9418
Triton X-100	MERCK	#T8787
BMP(14:0)2	Avanti Polar Lipids	#857131P
PE(14:0)2	Avanti Polar Lipids	#850745X
PS(14:0)2	Avanti Polar Lipids	#840033X
PC(14:0)2	Avanti Polar Lipids	#850345C
PG(14:0)2	Avanti Polar Lipids	#840445X
PA(14:0)2	Avanti Polar Lipids	#830845X
CL(14:0)4	Avanti Polar Lipids	#710332C
Lyso-PE(14:0)	Avanti Polar Lipids	#856735X
Lyso-PC(14:0)	Avanti Polar Lipids	#855575C
Lyso-PA(14:0)	Avanti Polar Lipids	#857120X
Lyso-PG(14:0)	Avanti Polar Lipids	#858120X
SM(12:0)	Avanti Polar Lipids	#860583P
TAG(14:0)3	Council of Europe EDQM	#T2500100
DAG(14:0)2	Cayman Chemicals	#15077
PI(8:0)2	Cayman Chemicals	#10008099
CE(16:0)-d7, 16:0	Avanti Polar Lipids	#700149
Ceramide/Sphingoid MIX I	Avanti Polar Lipids	#LM6002
ST(17:0)	Avanti Polar Lipids	#860572
Chloroform	Biosolve	#0003480702BS
Methanol	Biosolve	#0013683502BS
2-Propanol	Biosolve	#0016246102BS
Ammonia	Merck	#M5432

**Critical commercial assays**		

CytoTune™-iPSC 2.0 Sendai Reprogramming Kit	ThermoFisher	A16517

**Experimental models: Cell lines**		

Hvs88 (VUi031-A)	iPSC Core Facility at Amsterdam UMC	–
Hvs451 (Vui032-A)	iPSC Core Facility at Amsterdam UMC	–
Hvs420 (Vui036-A)	iPSC Core Facility at Amsterdam UMC	–
ALD001	iPSC Core Facility at Amsterdam UMC	–
ALD010	iPSC Core Facility at Amsterdam UMC	–
ALD014	iPSC Core Facility at Amsterdam UMC	–
ALD022	iPSC Core Facility at Amsterdam UMC	–
ALD025	iPSC Core Facility at Amsterdam UMC	–
ALD030	iPSC Core Facility at Amsterdam UMC	–
ALD212	iPSC Core Facility at Amsterdam UMC	–
ALD222	iPSC Core Facility at Amsterdam UMC	–
ALD307	iPSC Core Facility at Amsterdam UMC	–
ALD202	iPSC Core Facility at Amsterdam UMC	–

### Experimental model and study participant details

#### Ethical approval

Fibroblasts from patients were obtained from the PEROX Biobank (IRB #2015_066), part of a natural history study in ALD (IRB #2018-310). All patients provided written informed consent.

#### Generation of hiPSCs

Fibroblasts from 6 ALD patients were reprogrammed with a lentiviral construct overexpressing OCT4, SOX2, KLF-4, and C-MYC, and fibroblasts from 4 ALD patients were reprogrammed using the CytoTune-iPSC 2.0 Sendai Reprogramming Kit, containing Oct3/4, Sox2, Klf-4, and c-Myc. See Table S1 for an overview of the hiPSC lines. Generated hiPSC lines were confirmed for pluripotency by immunocytochemistry, PCR, alkaline phosphatase, embryoid body formation assay, karyotyping and/or a Pluritest. Quantitative PCR was performed to confirm the absence of Sendai viral sequences in iPSCs after 10 passages, verifying successful clearance of the reprogramming vector. Control and ALD hiPSCs were maintained on Vitronectin XF (Stem Cell Technologies, Vancouver, BC, Canada, #07180) coated plates in TeSR-E8 medium (Stem Cell Technologies, Vancouver, BC, Canada, #05990). Medium was refreshed daily, and cells were passaged once a week using Gentle Cell Dissociation Reagent (Stem Cell Technologies, #100–0485) according to manufacturer’s protocol. Cells were split in 1:10 to 1:50 ratios to a new well for further maintenance. All cell lines were tested regularly for mycoplasma contamination.

#### Cortical organoid generation

Cortical organoids were generated from hiPSCs as previously described^12^ with the following modifications. In short, hiPSC colonies cultured on vitronectin (Stem Cell Technologies, #07180) and in TeSR-E8 medium (Stem Cell Technologies, #05990) were dissociated with Accutase (MERCK, #SF006) at 37°C for 7 min, made into a single-cell suspension, and 9.000 cells were plated into individual wells of ultra-low attachment U-bottom 96-well plates (Corning, #CLS7007) in 150 μL of TeSR-E8 medium supplemented with ROCK inhibitor (10 μM; Selleckchem, #S1049) and FGF2 (4 ng/ml; Peprotech, #AF-100-18B). On day 2, 150 μL of TeSR-E8 medium supplemented with the two SMAD inhibitors dorsomorphin (2.5 μM; Selleckchem, #S7306) and SB431542 (10 μM; Selleckchem, #S1067) was added. Two-thirds of this medium was refreshed daily for the next five days. On day 7, medium was changed to Neural Maintenance Medium (NMM) consisting of 1:1 DMEM/F12 (Thermo Fisher Scientific, #11530566) and Neurobasal Medium (Thermo Fisher Scientific, #11570556) supplemented with 0.5% N2 (Thermo Fisher Scientific, #15410294), 1% B27-A (Thermo Fisher Scientific, #15440584), human insulin (5 μg/ml; MERCK, #I9278), L-glutamine (1.5 mM; Thermo Fisher Scientific, #11500626), non-essential amino acids (100 μM; Thermo Fisher Scientific, #11140035), β-mercaptoethanol (10 μM; Thermo Fisher Scientific, #21985023), and penicillin-streptomycin (100U/ml; Thermo Fisher Scientific, #15140122), and supplemented with EGF (20 ng/mL; Peprotech, #AF-10015), FGF-2 (10 ng/mL). From day 7 onwards, medium was refreshed every other day. On day 25, organoids were moved to Poly(2-hydroxyethyl methacrylate)-treated (MERCK, #P3932) 24-well plates (VWR, #391–3370), one organoid per well, in NMM supplemented with NT3 (20 ng/ml; Peprotech, #450-03), BDNF (20 ng/ml; Peprotech, #450-02), cAMP (1 μM; MERCK, #D0260), and Ascorbic acid (150 μM; MERCK, #A4544), with three-quarters of medium refreshments every three to four days. On day 50, NMM was supplemented with PDGF (10 ng/ml; Bio-techne, #221-AA), IGF1 (10 ng/ml; Peprotech, #100-11), T3 (60 ng/ml; MERCK, #T6397), cAMP (1 μM), and Ascorbic acid (150 μM). From day 70, NMM was not supplemented with PDGF, and media remained the same until the end of the protocol.

#### Spinal cord organoid generation

Spinal cord organoids were generated from hiPSCs as previously described^13^ with the following modifications. hiPSC colonies were cultured and dissociated as described in cortical organoid generation. On day 2, 150 μL of TeSR-E8 medium supplemented with the two SMAD inhibitors dorsomorphin (2.5 μM) and SB431542 (10 μM) was added. Two-thirds of this medium was refreshed daily for the next five days, supplementing with CHIR 99021 (3 μM; Cayman Chemicals, #13122) from day 6–20. On day 7, medium was changed to Neural Maintenance Medium (NMM) consisting of 1:1 DMEM/F12 and Neurobasal Medium supplemented with 0.5% N2, 1% B27 (Thermo Fisher Scientific, #15360284), human insulin (5 μg/ml), L-glutamine (1.5 mM), non-essential amino acids (100 μM), β-mercaptoethanol (10 μM), and penicillin-streptomycin (100U/ml), and supplemented with EGF (20 ng/mL), FGF-2 (10 ng/mL), and RA (0.1 μM, ReproCell, #04–0021) until day 20, with the addition of SAG (0.1 μM; Cayman Chemicals, #11914) from day 13. From day 8 on, medium was refreshed every other day. On day 21, spinal cord organoids were moved to Poly(2-hydroxyethyl methacrylate)-treated 24-well plates, one organoid per well, in NMM supplemented with NT3 (20 ng/ml), BDNF (20 ng/ml), IGF1 (10 ng/ml), cAMP (1 μM), and Ascorbic acid (150 μM; MERCK, #A4544), with three-quarters of medium refreshments every three to four days. On day 35 onward, spinal cord organoids were maintained in NMM supplemented with PDGF (10 ng/ml), IGF1 (10 ng/ml), T3 (60 ng/ml), cAMP (1 μM), and Ascorbic acid (150 μM). From day 55 NMM was not supplemented with PDGF, and media remained the same until the end of the protocol. Due to an unexpected contamination, spinal cord organoids were only characterized up to 100 days, while cortical organoids were characterized up to 200 days.

### Method details

#### Lipidomics

Organoids were pooled in an Eppendorf and washed with PBS. PBS was removed and organoids were stored at −80°C. Per donor and timepoint, three samples were used for lipidomics. Each sample consisted of a pool of organoids from the same donor to ensure sufficient input (day 50: 3 organoids; day ≥100: 2 organoids). Lipidomics analysis and bioinformatics was performed as previously described.^29^ In a 2 mL tube 200 μg was added and mixed with a mix of internal standards for different lipid classes, including 0.1 nmol of cardiolipin CL(14:0/14:0/14:0/14:0), 2.0 nmol of phosphatidylcholine PC(14:0/14:0), 0.1 nmol of phosphatidylglycerol PG(14:0/14:0), 5.0 nmol of phosphatidylserine PS(14:0/14:0), 0.5 nmol of phosphatidylethanolamine PE(14:0/14:0), 0.5 nmol of phosphatidic acid PA(14:0/14:0), 2.125 nmol of sphingomyelin SM(d18:1/12:0), 0.02 nmol of lysophosphatidylglycerol LPG(14:0), 0.1 nmol of lysophosphatidylethanolamine LPE(14:0), 0.5 nmol of lysophosphatidylchloline LPC(14:0), 0.1 nmol of lysophosphatidic acid LPA(14:0), 0.5 nmol of phosphatidylinositol PI(8:0/8:0), 0.5 nmol diglycerides DG(14:0/14:0), 0.5 nmol triglycerides TG(14:0/14:0/14:0), 2.5 nmol cholesterol ester D7-CE(16:0), 0.125 nmol of sphingosine and ceramide mix (Avanti Polar Lipids) dissolved in 1:1 (v/v) methanol:chloroform. Next, 1.5 mL 1:1 (v/v) methanol:chloroform was added to each sample. The mixture was sonicated in a water bath (5 min) and centrifuged (4 °C, (16,000×g, 10 min). The supernatant was transferred to a 1.5 mL glass auto sampler vial and evaporated under a stream of nitrogen at 45°C. The dried lipids were reconstituted in 100 μL of 1:1 (v/v) chloroform:methanol. Chromatographic separation of lipids was done using a Thermo Fisher Scientific Ultimate 3000 binary UPLC using a normal phase and a reverse phase column in separate runs. Normal-phase separation was done using a Phenomenex LUNA silica, 250 × 2 mm, 5 μm 100 Å column. Column temperature was held constant at 25°C. The composition of the mobile phase A consisted of 85:15 (v/v) methanol:water containing 0.0125% formic acid and 3.35 mmol/L ammonia and the composition of mobile phase B consisted of 97:3 (v/v) chloroform:methanol containing 0.0125% formic acid. The LC gradient started at of 10% A for 0–1 min, 20% A at 4 min, 85% A at 12 min, 100% A at 12.1 min, 100% A for 12.1–14 min, 10% A at 14.1 min, 10% A for 14.1–15 min using a flow rate of 0.3 mL/min. Reversed-phase separation was done using a Waters HSS T3 column (150 × 2.1 mm, 1.8 μm particle size). The composition of the mobile phase A consisted of 4:6 (v/v) methanol:water and B 1:9 (v/v) methanol:isopropanol, both containing 0.1% formic acid and 10 mmol/L ammonia. The gradient started at 100% A going to 80% A at 1 min and 0% A at 16 min, 0% A for 16–20 min, 100% A at 20.1 min and 100% A for 20.1–21 min. The column temperature was held constant at 60°C and a flow rate of 0.4 mL/min was used. After LC separation, lipids were detected using a Q Exactive Plus Orbitrap mass spectrometer (Thermo Scientific) using negative and positive ionization. The spray voltage was 2,500 V and nitrogen was used as the nebulizing gas. A resolution of 280.000 was used in a mass range of m/z 150 to m/z 2.000.

#### Lipidomics data analysis and statistics

The raw LC/MS data were converted to mzXML format using MSConvert.^43^ Lipidomics data processing and analysis were done as described earlier.^29^ Lipidomics data were analyzed using an in-house developed lipidomics pipeline based on the R programming language (http://ww.r-project.org) and MATLAB. Preprocessing was done using the R package XCMS with minor changes to some functions to better suit the Q Exactive data; notably, the definition of noise level in centWave was adjusted, and the stepsize in fillPeaks.^44^ Lipid identification was performed using a combination of accurate mass measurements, relative retention times, injection of relevant standards, and analysis of samples with known metabolic defects. Lipid classes were defined by their generic chemical formulas, where ‘R’ denotes the radyl group. Upon importing the lipid database into the annotation pipeline, the generic chemical formula for each lipid class was expanded by replacing the ‘R’ element with a range of potential radyl group lengths and degrees of unsaturation. This expanded list of chemical formulas was then used to calculate the neutral monoisotopic mass of each species. The monoisotopic mass was then converted into a set of m/z values corresponding to each adduct and charge combination, allowing reliable measurement and annotation of the lipid species. The reported lipid abundances are semi-quantitative, calculated by dividing the analyte response (peak area) by that of the corresponding internal standard, with the result further adjusted for the concentration of the internal standard, and expressed in arbitrary units (A.U.). Group differences in the lipidomics data were tested for statistical significance with a linear mixed-effects model (LMM) on the log transformed data, where subject-specific deviations from the group mean were modeled with random intercepts. Visualization of lipidomics data was done using median values of three replicate samples per donor and timepoint. Error bars reflect variability across donors.

#### Immunohistochemistry

Briefly, organoids were washed with PBS, fixed in 4% paraformaldehyde (PFA in dH_2_O, Electron Microscopy Sciences, #15710) at room temperature for 30 min, and followed by sucrose cryopreservation (30% sucrose in PBS at 4°C for 48h), embedding in OCT (Tissue-Tec Oct Compound, #4583), and snap-freezing. For IHC, 20 μm thick sections were cut using a cryostat (Leica). After 6× PBS washes of 5 min, antigen retrieval was performed by incubating slides in 0.01M citrate buffer at 90°C for 30 min. After one PBS wash, slides were blocked for 1h at room temperature with blocking buffer consisting of PBS + goat serum (5%; Thermo Scientific, #16210) + BSA (0.1%; MERCK, #A9418) + Triton X-100 (0.3%, MERCK, #T8787). Primary antibodies in blocking buffer were incubated 1h at room temperature and then overnight at 4°C. The next day, after 6× PBS washes of 5 min, organoids were incubated with secondary antibodies (1:1000; Thermo Scientific, Alexa Fluor 488, 594, 647) for 2 h at room temperature. Afterward, cells were washed 6 × 5 min with PBS, incubated with diamidino-2-phenylindole (1:1000; DAPI; MERCK, #D9542) for 2 min at room temperature, washed once with PBS and embedded with Fluoromount-G (Southern Biotech, #0100-01).

#### Organoid imaging and analysis

Confocal images of organoids were acquired with a Nikon ECLIPSE Ti inverted microscope (Nikon Corporation, Tokyo, Japan) controlled by NIS-Elements 4.30 software (Nikon Corporation) and fluorescent images of motor neurons were acquired with a Leica DMi8 inverted light microscope (Leica Microsystems) controlled by LAS X 3.7 software (Leica Microsystems).

OLIG2, O4, and MBP-positive cells in organoids were analyzed using CLIJ2 plugin for Fiji. Briefly, within CLIJ2 positive cells for the different markers in organoids were labeled by applying a subtracting a Gaussian Blur2D filter > Thresholding > Connected components labeling Box > Duplicate and go ahead in Fiji. Next, positive cells were quantified in Fiji. All analyses were performed blinded. We used GraphPad Prism 9.0 for statistical analysis.

Fluorescent images of organoid sections were acquired using an Olympus SLIDEVIEW VS200 slide scanner (Evident Scientific, Tokyo, Japan) controlled by OlyVIA software (Evident Scientific). Quantification of positive staining area for cleaved caspase-3 (CC3), phospho-histone H3 (PH3), SATB2, MAP2, and neurofilament (NF) was performed using FIJI software by applying intensity thresholding and measuring the area after thresholding, calculated as percentage of organoid section area.

#### Electron microscopy

Organoids were fixed for 90 min at RT with 2.5% glutaraldehyde in 0.1 M cacodylate buffer, pH 7.4. After fixation, organoids were washed three times for 5 min with 0.1 M cacodylate buffer, pH 7.4, postfixed for 1 h at RT with 1% OsO_4_/1% KRu(CN)_6_. After dehydration through a series of increasing ethanol concentrations, organoids were embedded in EPON and polymerized for 48 h at 60°C in a flat embedding mold. Ultrathin sections (80 nm) were collected on copper mesh grids and stained in uranyl acetate and lead citrate in an automated stainer (Leica EM AC20). Images were collected on a JEM1010 transmission electron microscope (JEOL) equipped with side-mounted CCD camera (Modera, Olympus/EMsis) at 60 kV.

### Quantification and statistical analysis

Data are presented as mean ± S.E.M. unless otherwise indicated. Distribution of the raw data was tested for normality distribution. Specifics on the statistical test applied for each experiment are included in the figure legends and methods section. In general, *n* values refer to the number of individual donor lines, and details are provided for each experiment in the figure legends. *p* values of immunohistochemical quantification comparing control and ALD groups were calculated using two-tailed nested *t* test.
