# Supplementary material for: Evolution of the lipidome uncovers early changes in adrenoleukodystrophy human cortical and spinal organoids
Source: iScience. 2025 Dec 4;29(1):114339. doi: 10.1016/j.isci.2025.114339 (PMC12774698; doi:10.1016/j.isci.2025.114339)
Supplement: Document S1. Figures S1–S16 and Table S1 [file mmc1.pdf]

iScience, Volume *29*

Supplemental information

Evolution of the lipidome uncovers early changes in adrenoleukodystrophy human cortical and spinal organoids

Roberto Montoro Ferrer, Yorrick R.J. Jaspers, Nicki Coveña, Nicole Breeuwsma, Inge M.E. Dijkstra, Julia Kempff, Jan-Bert van Klinken, Joke Wortel, Jan R.T. van Weering, Marc Engelen, Stephan Kemp, and Vivi M. Heine

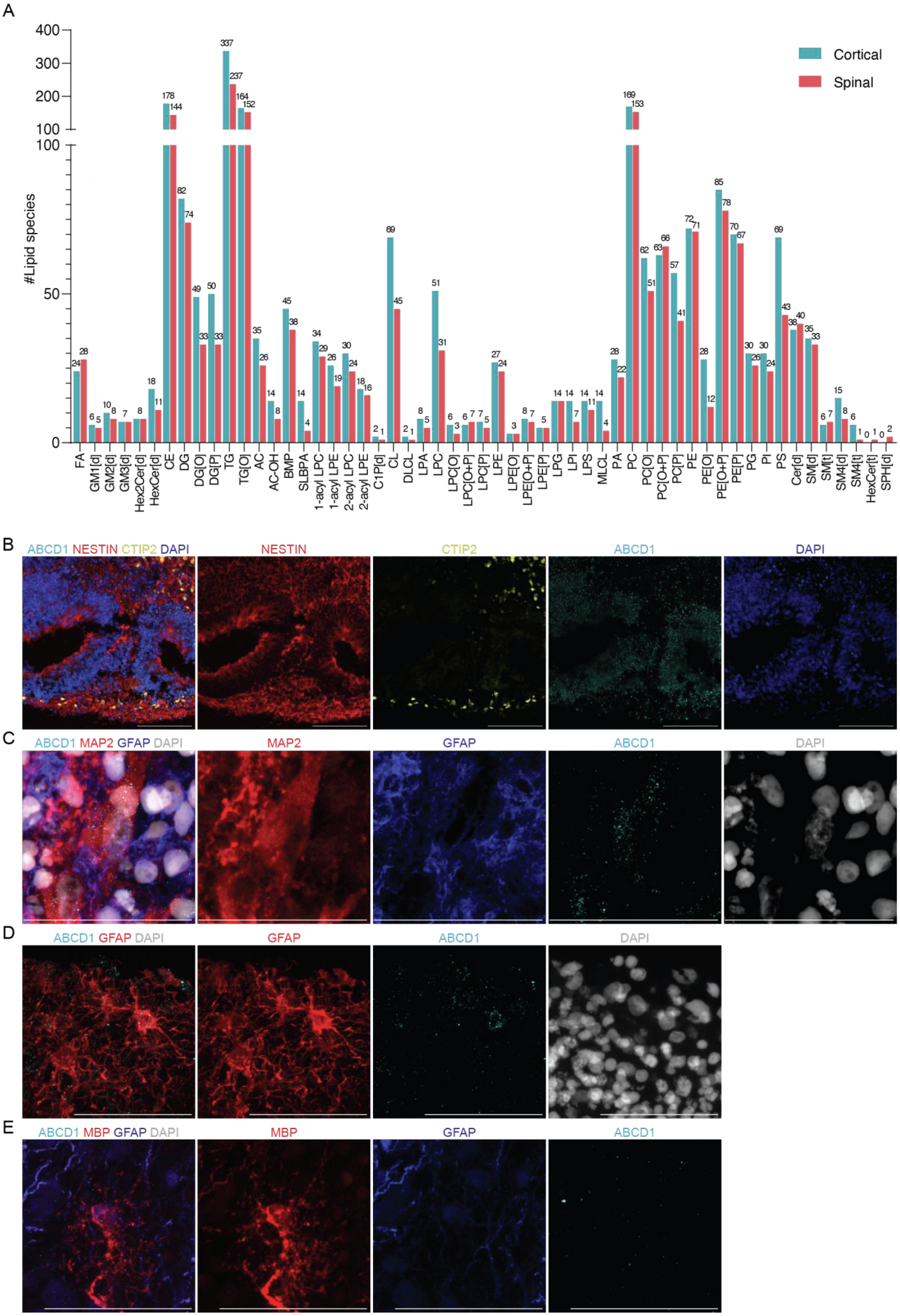
A
#Lipid species
Cortical
Spinal
FA
GM1[d]
GM2[d]
GM3[d]
Hex2Cer[d]
HexCer[d]
CE
DG
DG[O]
DG[P]
TG
TG[O]
AC
AC-OH
BMP
SLBPA
1-acyl LPC
1-acyl LPE
2-acyl LPC
2-acyl LPE
C1P[d]
CL
DLCL
LPA
LPC
LPC[O]
LPC[O+P]
LPC[P]
LPE
LPE[O]
LPE[O+P]
LPE[P]
LPG
LPI
LPS
MLCL
PA
PC
PC[O]
PC[O+P]
PC[P]
PE
PE[O]
PE[O+P]
PE[P]
PG
PI
PS
Cer[d]
SM[d]
SM[t]
SM4[d]
SM4[t]
HexCer[t]
SPH[d]
B
ABCD1 NESTIN CTIP2 DAPI
NESTIN
CTIP2
ABCD1
DAPI
C
ABCD1 MAP2 GFAP DAPI
MAP2
GFAP
ABCD1
DAPI
D
ABCD1 GFAP DAPI
GFAP
ABCD1
DAPI
E
ABCD1 MBP GFAP DAPI
MBP
GFAP
ABCD1

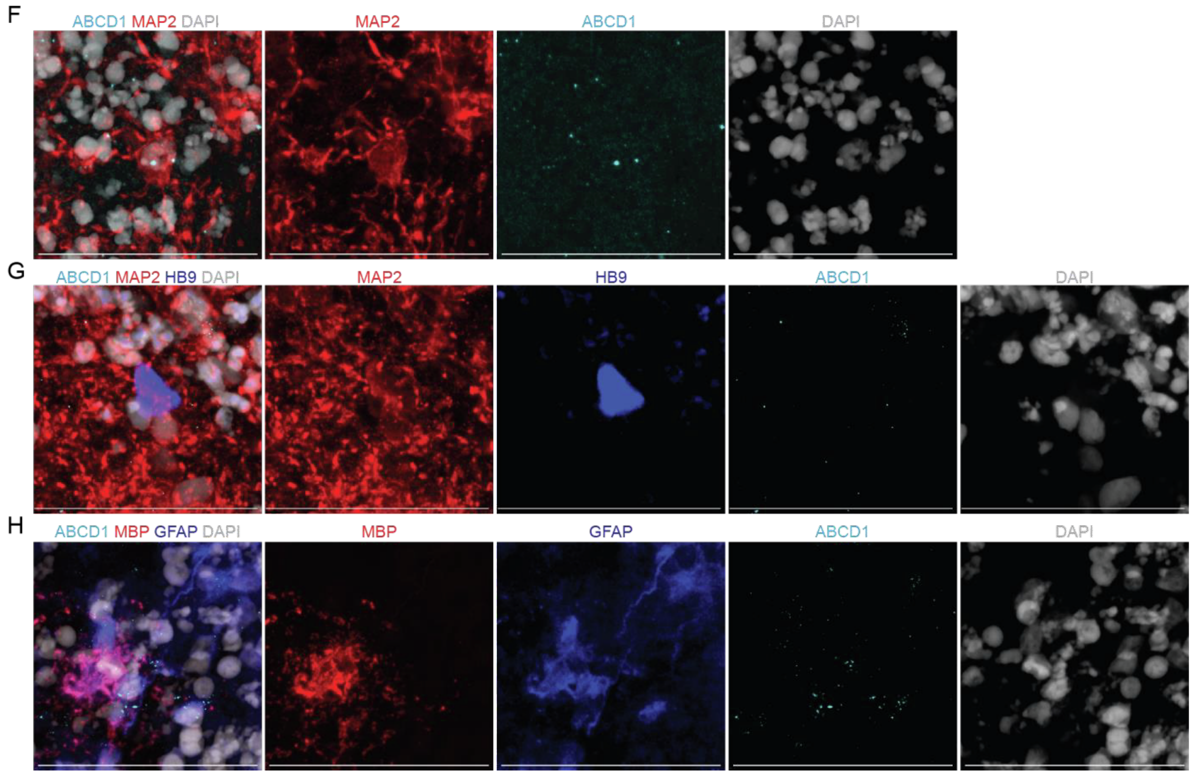

Figure S1

(A) Overview of identified lipids covering 53 lipid subclasses and more than 2,200 lipid species. Bars represent the number of detected lipid species for each lipid class, with cortical and spinal cord organoids compared side by side. Numbers above the bars indicate the number of lipid species detected within each lipid class.

(B) Representative confocal immunofluorescence image of ABCD1 present throughout the ventricular-like zones and CTIP2$^+$ cortical-like plate at day 50 (scale bar 50µm).

(C-E) Single channel images corresponding to Figure 1F (scale bar 50µm).

(F-H) Single channel images corresponding to Figure 1J (scale bar 50µm).

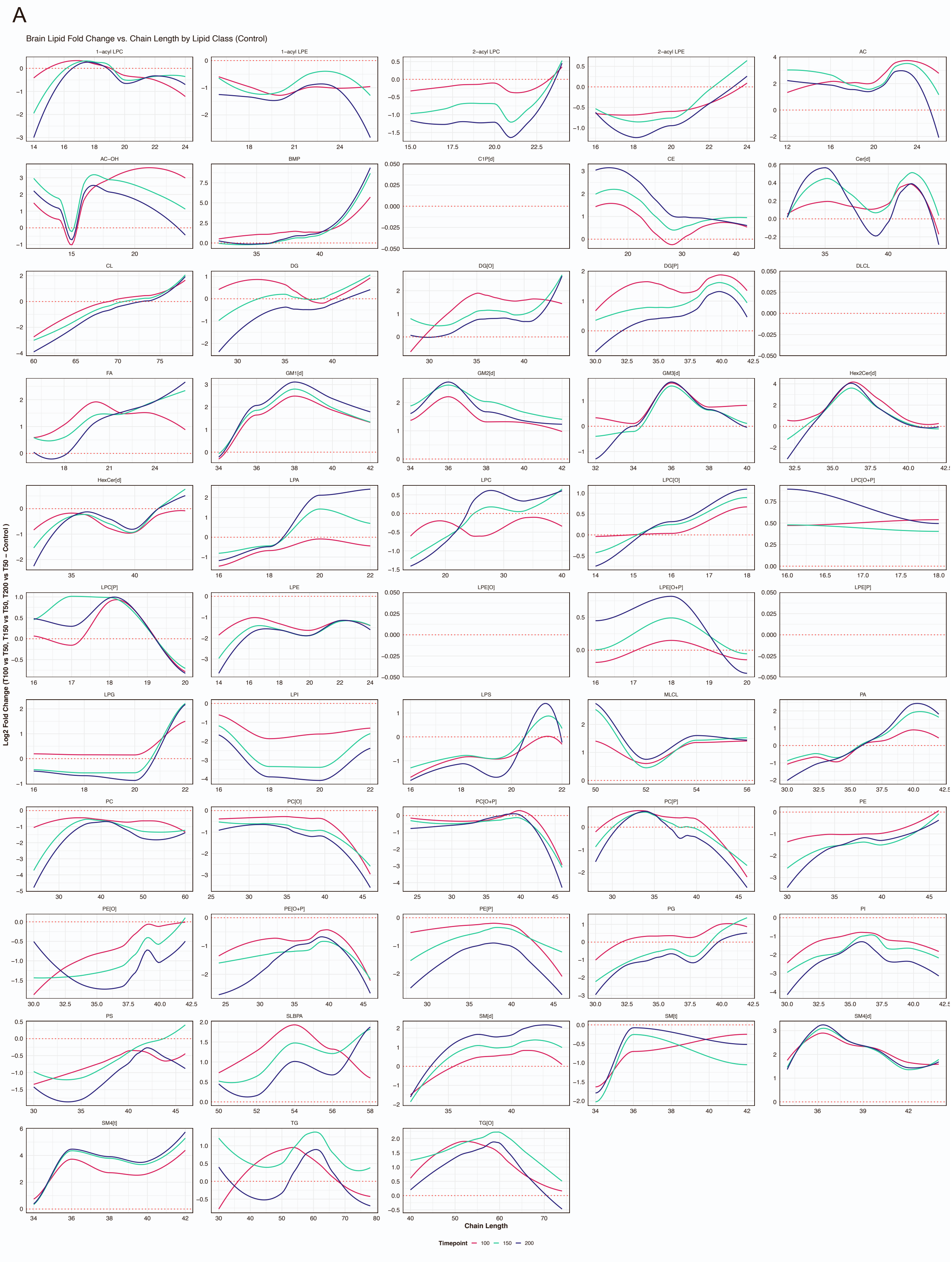

Figure S2

(A) Log2 fold changes of lipid classes as a function of total acyl chain length of hCO across timepoints day 100, 150, 200, relative to day 50.

A

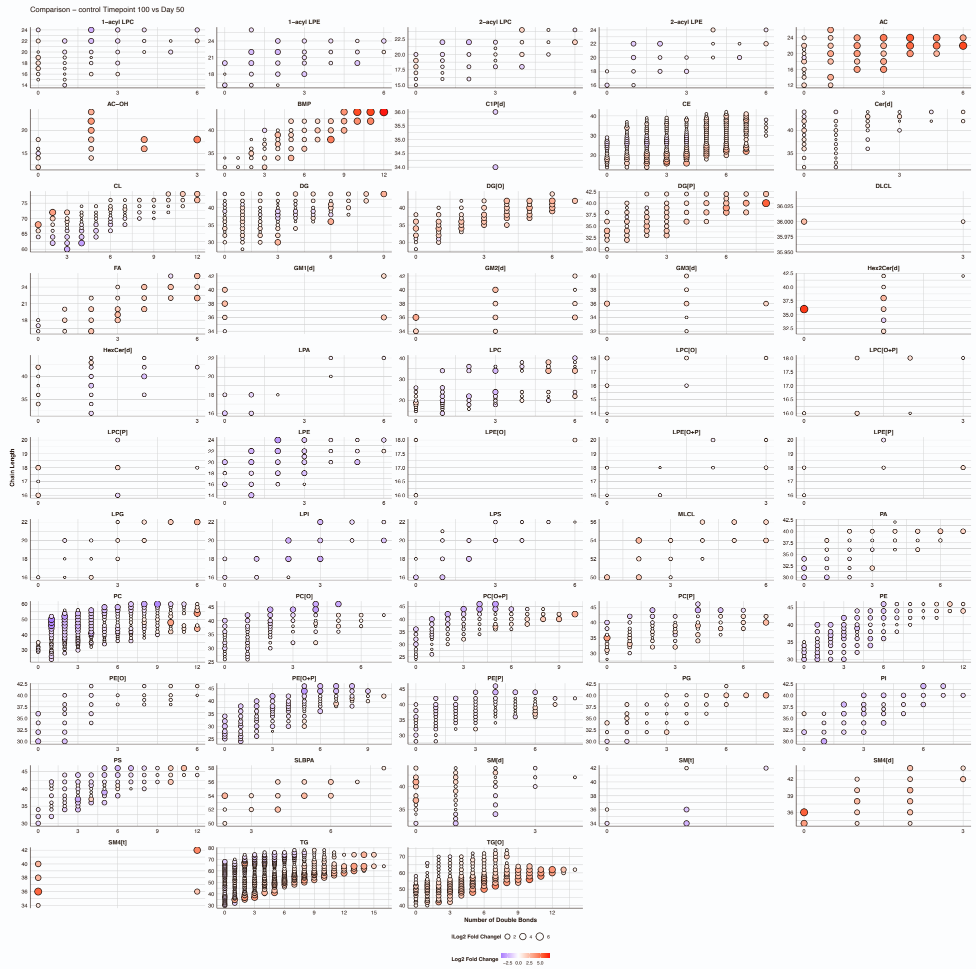

Comparison – control Timepoint 100 vs Day 50
1–acyl LPC
1–acyl LPE
2–acyl LPC
2–acyl LPE
AC
AC–OH
BMP
C1P[d]
CE
Cer[d]
CL
DG
DG[O]
DG[P]
DLCL
FA
GM1[d]
GM2[d]
GM3[d]
Hex2Cer[d]
HexCer[d]
LPA
LPC
LPC[O]
LPC[O+P]
LPC[P]
LPE
LPE[O]
LPE[O+P]
LPE[P]
LPG
LPI
LPS
MLCL
PA
PC
PC[O]
PC[O+P]
PC[P]
PE
PE[O]
PE[O+P]
PE[P]
PG
PI
PS
SLBPA
SM[d]
SM[t]
SM4[d]
SM4[t]
TG
TG[O]
Chain Length
Number of Double Bonds
|Log2 Fold Change|
Log2 Fold Change

B

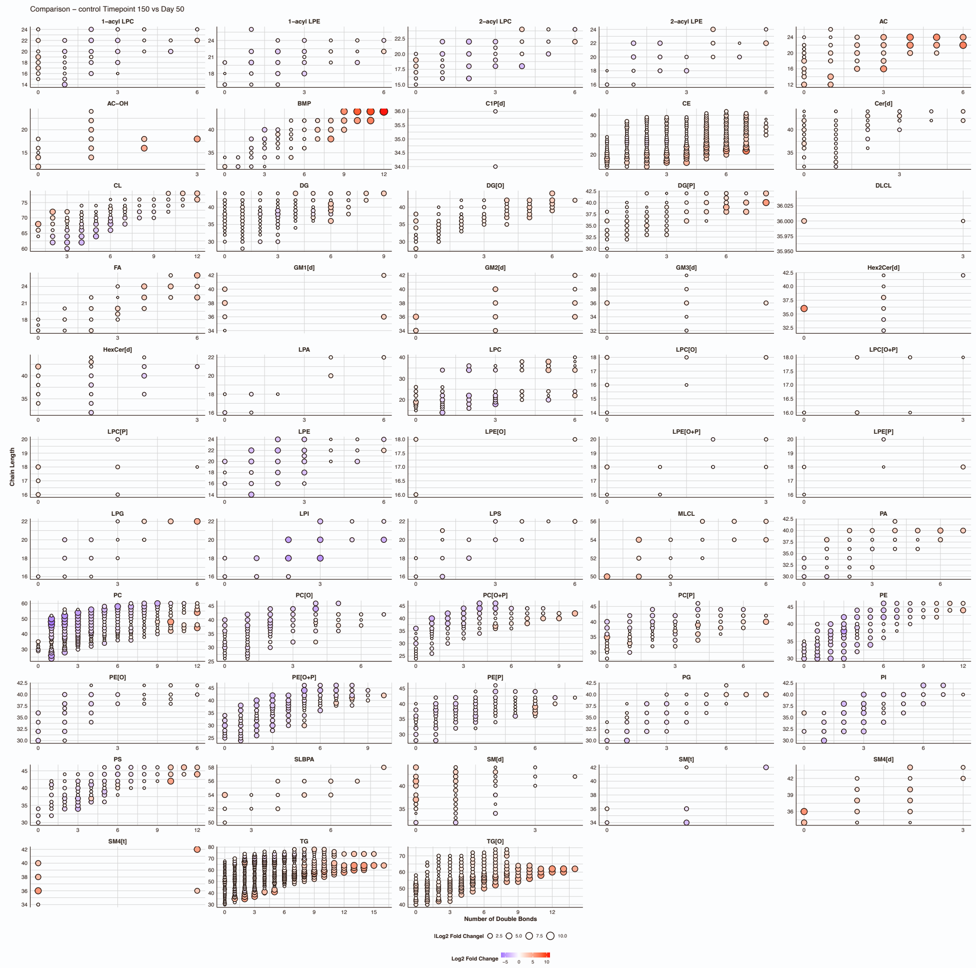

Comparison – control Timepoint 150 vs Day 50
1–acyl LPC
1–acyl LPE
2–acyl LPC
2–acyl LPE
AC
AC–OH
BMP
C1P[d]
CE
Cer[d]
CL
DG
DG[O]
DG[P]
DLCL
FA
GM1[d]
GM2[d]
GM3[d]
Hex2Cer[d]
HexCer[d]
LPA
LPC
LPC[O]
LPC[O+P]
LPC[P]
LPE
LPE[O]
LPE[O+P]
LPE[P]
LPG
LPI
LPS
MLCL
PA
PC
PC[O]
PC[O+P]
PC[P]
PE
PE[O]
PE[O+P]
PE[P]
PG
PI
PS
SLBPA
SM[d]
SM[t]
SM4[d]
SM4[t]
TG
TG[O]
Chain Length
Number of Double Bonds
|Log2 Fold Change|
Log2 Fold Change

C

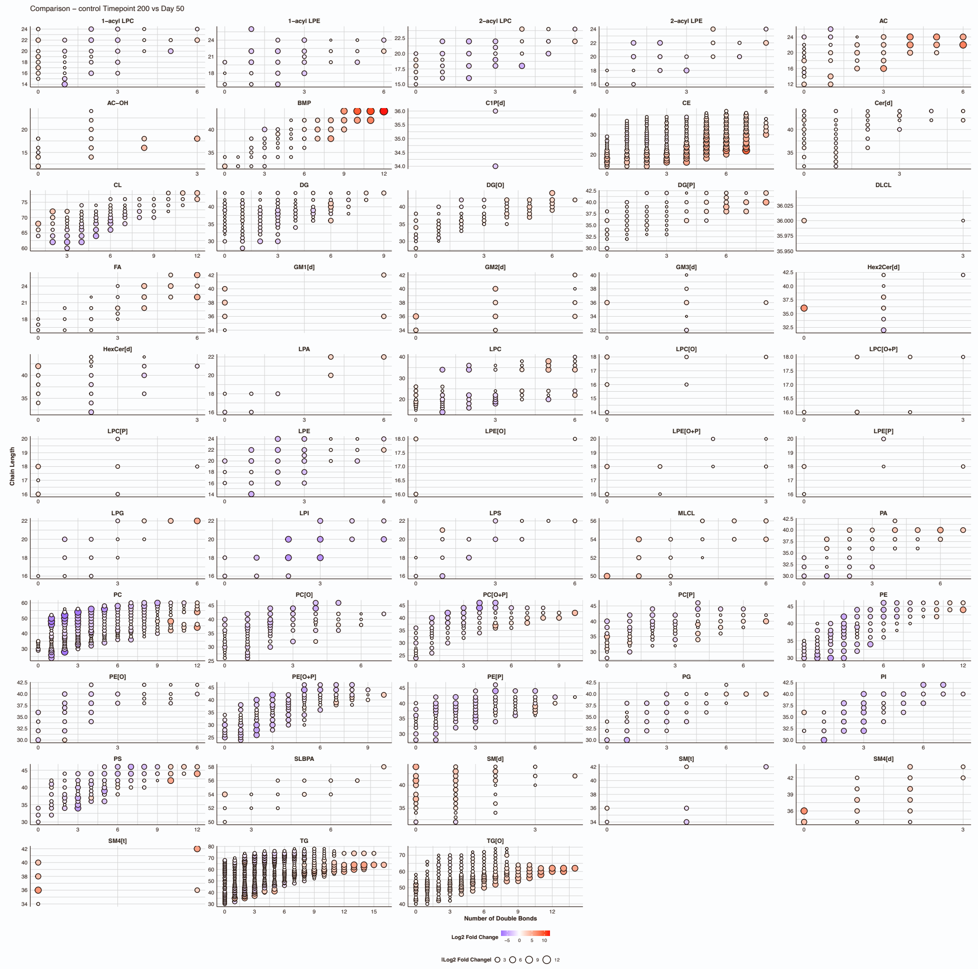

Figure S3

(A) Bubble plots illustrating the relationship between acyl chain length and the number of double bonds for lipid classes in control hCO, comparing day 100 to day 50.

(B) Bubble plots illustrating the relationship between acyl chain length and the number of double bonds for lipid classes in control hCO, comparing day 150 to day 50.

(C) Bubble plots illustrating the relationship between acyl chain length and the number of double bonds for lipid classes in control hCO, comparing day 200 to day 50. Bubble size corresponds to -Log10(p-value) and color intensity indicates Log2 fold change.

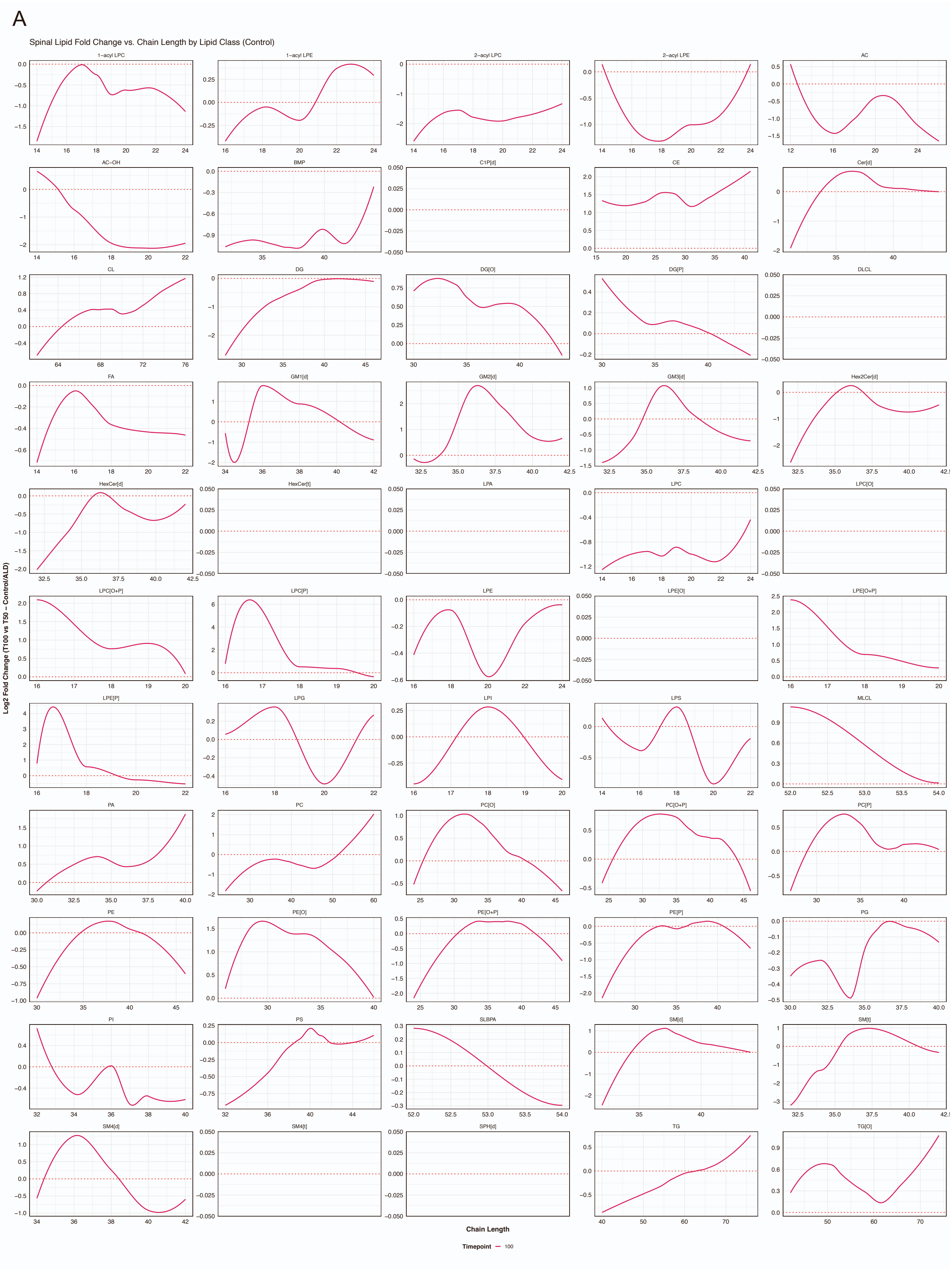
A
Spinal Lipid Fold Change vs. Chain Length by Lipid Class (Control)
1–acyl LPC
1–acyl LPE
2–acyl LPC
2–acyl LPE
AC
AC–OH
BMP
C1P[d]
CE
Cer[d]
CL
DG
DG[O]
DG[P]
DLCL
FA
GM1[d]
GM2[d]
GM3[d]
Hex2Cer[d]
HexCer[d]
HexCer[t]
LPA
LPC
LPC[O]
LPC[O+P]
LPC[P]
LPE
LPE[O]
LPE[O+P]
LPE[P]
LPG
LPI
LPS
MLCL
PA
PC
PC[O]
PC[O+P]
PC[P]
PE
PE[O]
PE[O+P]
PE[P]
PG
PI
PS
SLBPA
SM[d]
SM[t]
SM4[d]
SM4[t]
SPH[d]
TG
TG[O]
Log2 Fold Change (T100 vs T50 – Control/ALD)
Chain Length
Timepoint 100

Figure S4

A

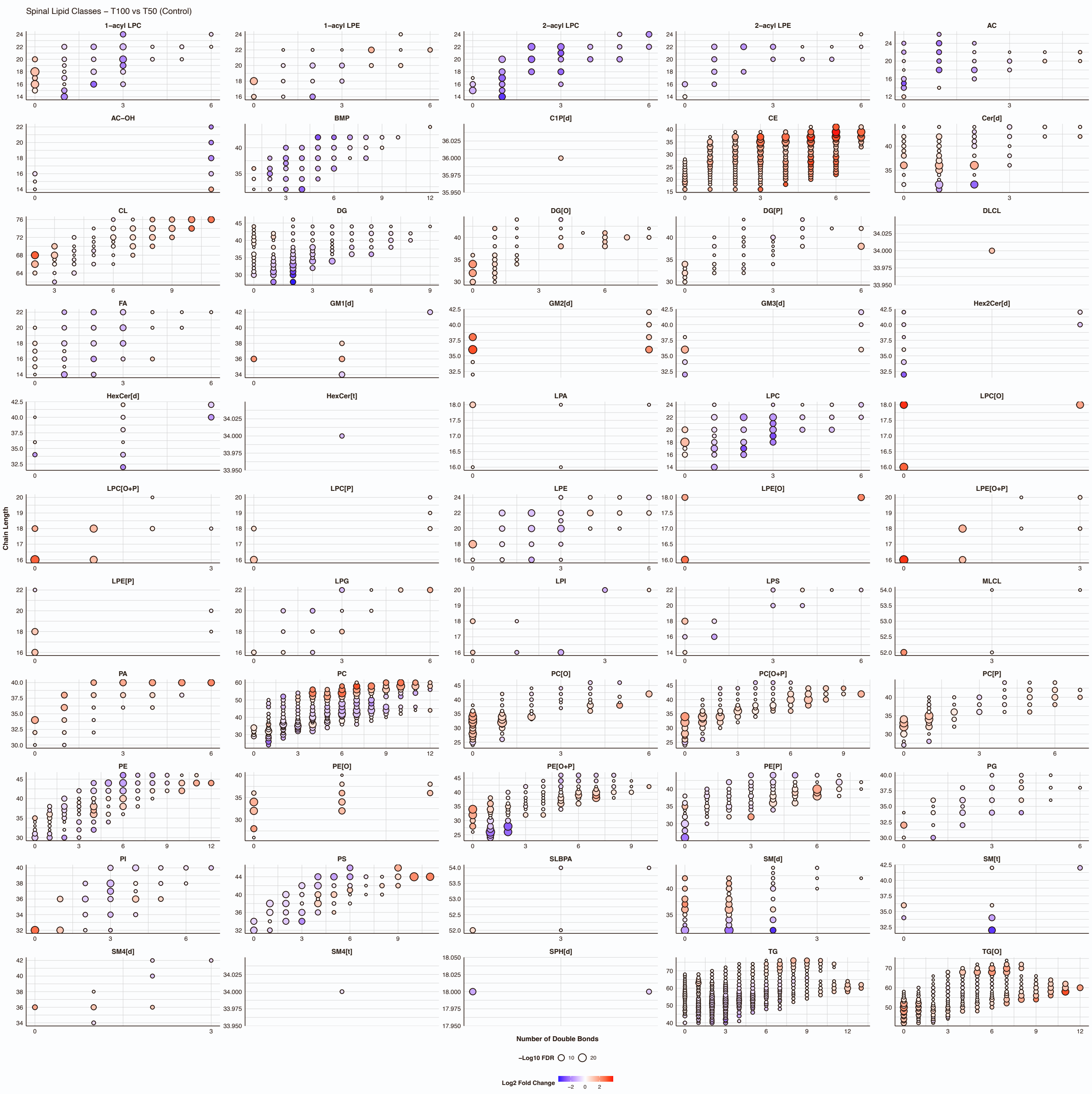

(A) Log2 fold changes of lipid classes as a function of total acyl chain length of hSO across timepoints day 100, relative to day 50.

Figure S5

(A) Bubble plots illustrating the relationship between acyl chain length and the number of double bonds for lipid classes in control hSO, comparing day 100 to day 50

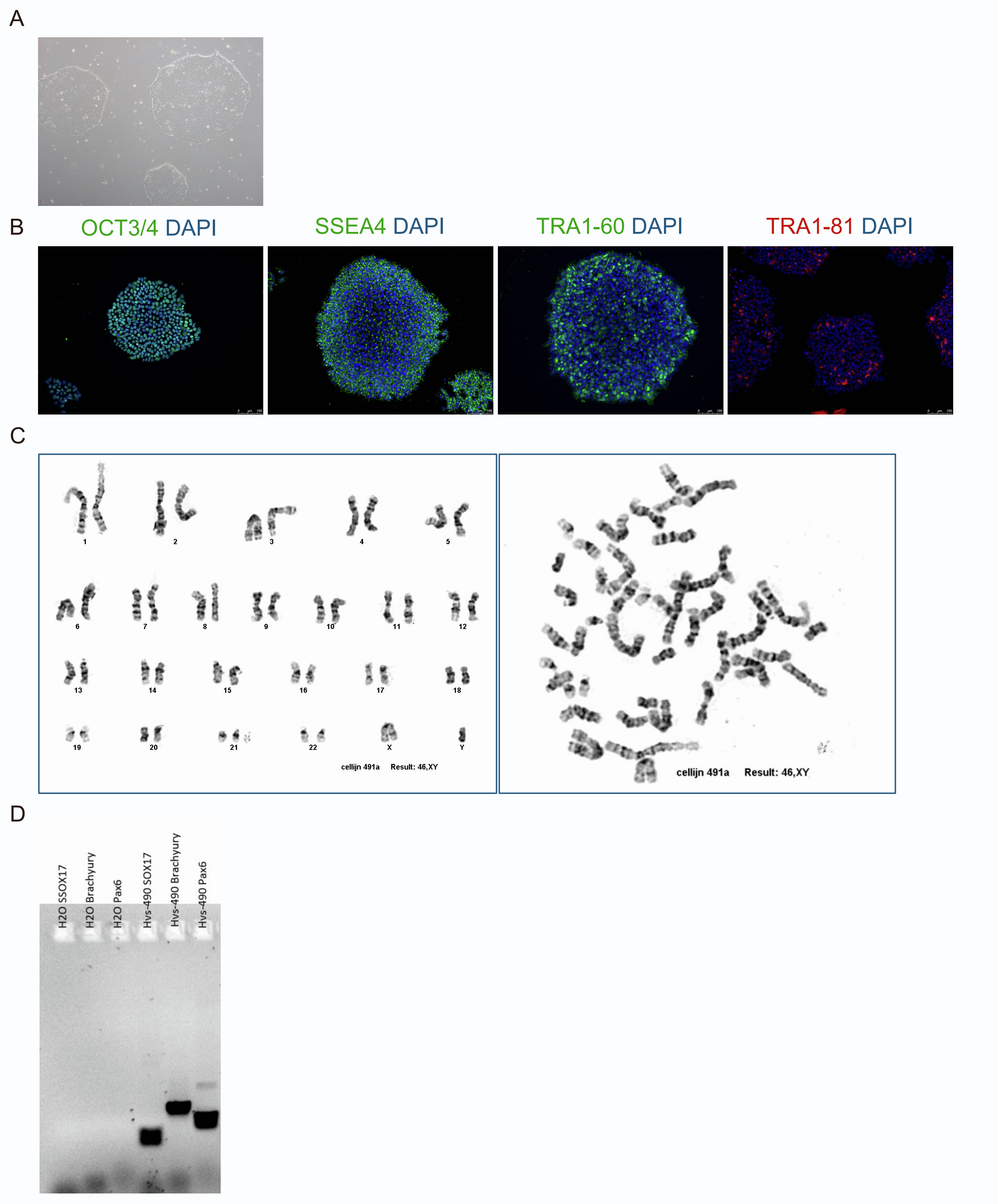

Figure S6

(A) Representative bright field picture of ALD hiPSC colonies.

(B) Representative immunofluorescent images of hiPSC colonies from an ALD line stained for the pluripotency markers OCT3/4, SSEA4, TRA1-60, and TRA1-80.

(C) Representative karyotyping of an ALD hiPSC line showing a correct karyotype.

(D) Representative gel showing cDNA bands corresponding to the three germ layer markers SOX17, Brachyury, and Pax6, indicative of a successful three germ layer differentiation.

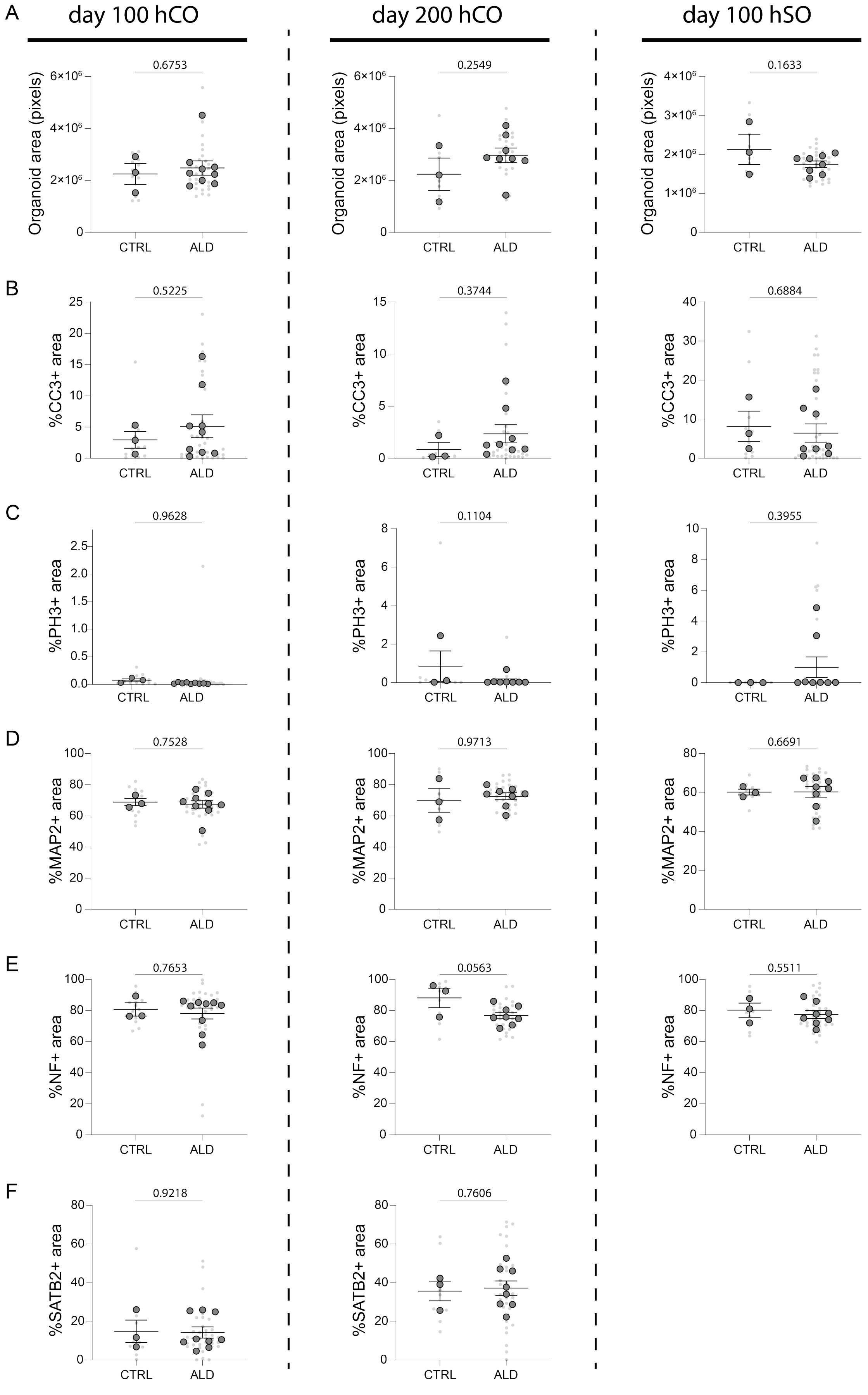

A
day 100 hCO
day 200 hCO
day 100 hSO
Organoid area (pixels)
0.6753
0.2549
0.1633
B
%CC3+ area
0.5225
0.3744
0.6884
C
%PH3+ area
0.9628
0.1104
0.3955
D
%MAP2+ area
0.7528
0.9713
0.6691
E
%NF+ area
0.7653
0.0563
0.5511
F
%SATB2+ area
0.9218
0.7606
CTRL
ALD

Figure S7

(A-F) Quantification of organoid area, and percentage of area coverage for markers Cleaved caspase 3, PH3, MAP2, and NF at day 100 and 200 hCO and at day 100 hSO, and for the marker SATB2 at day 100 and 200 hCO. Quantifications were performed in 3 whole tissue sections from 3-4 organoids per hiPSC line. *P* values were calculated using two-tailed nested t-test.

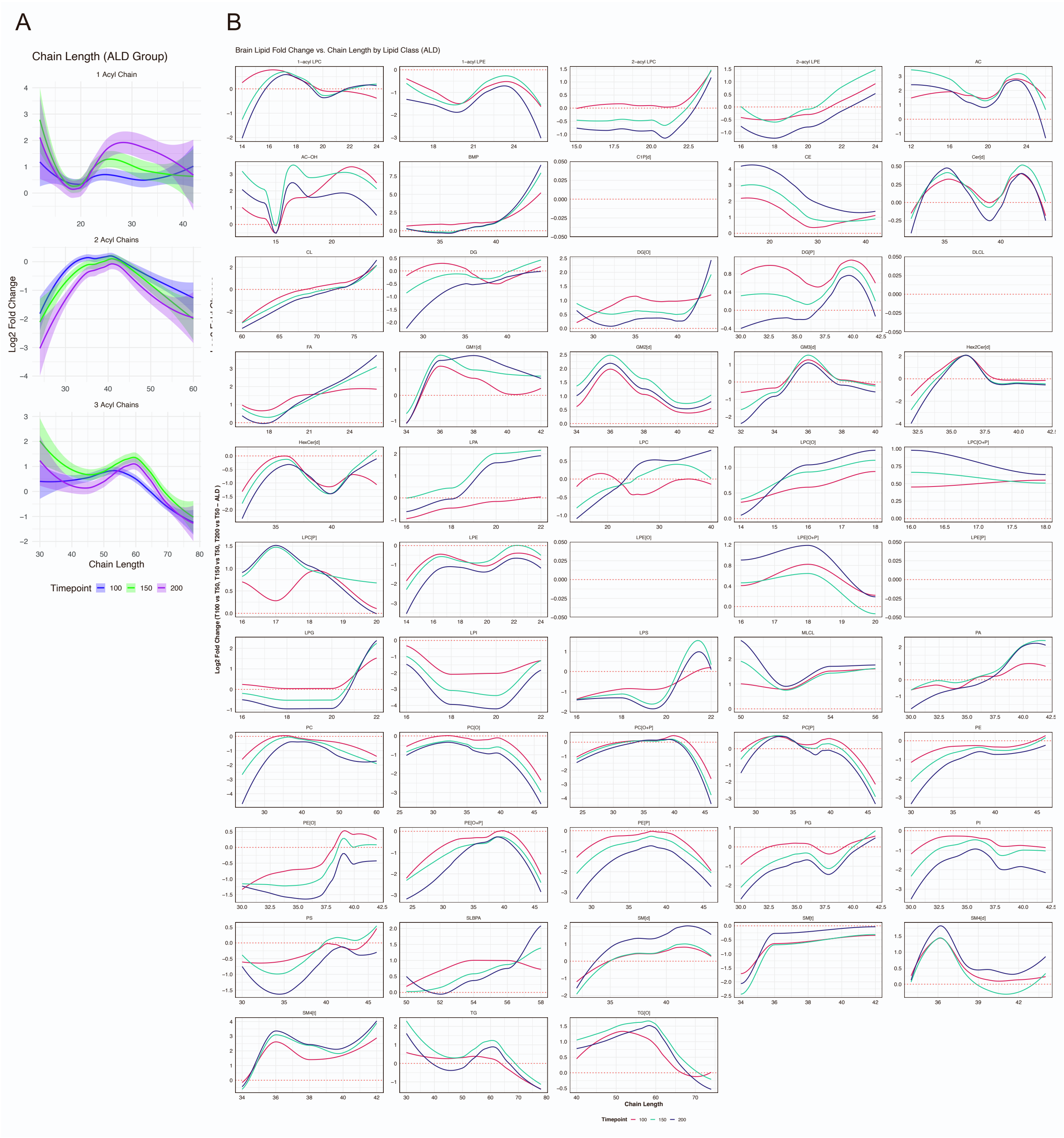

Figure S8

(A) Log2 fold changes of lipid classes with 1, 2, and 3 acyl chains as a function of total acyl chain length across timepoints for ALD hCO (day 100, 150, 200, relative to day 50).

(B) Log2 fold changes of lipid classes as a function of total acyl chain length of ALD hCO across timepoints day 100, 150, 200, relative to day 50.

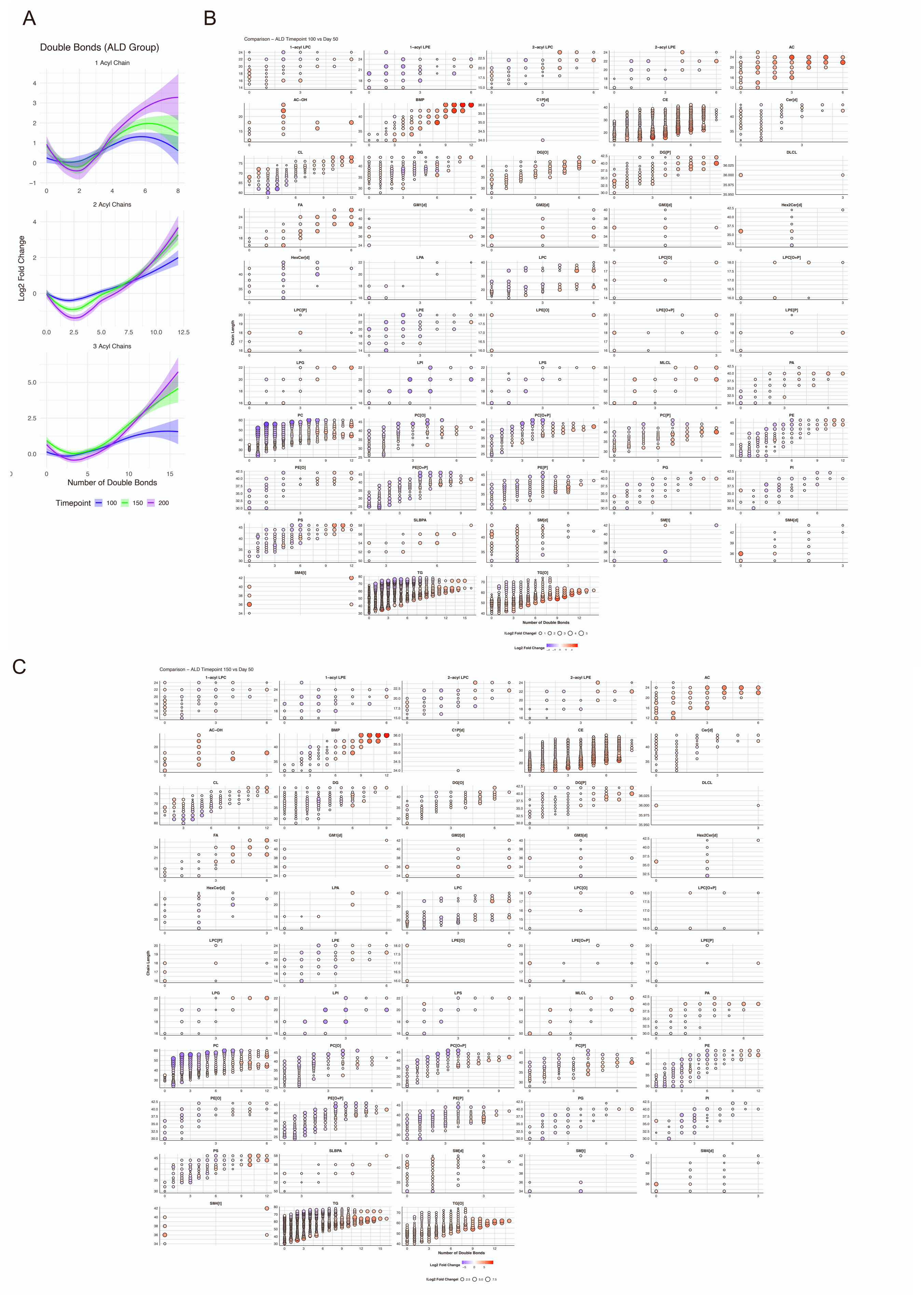
A
Double Bonds (ALD Group)
1 Acyl Chain
2 Acyl Chains
3 Acyl Chains
Log2 Fold Change
Number of Double Bonds
Timepoint 100 150 200
B
Comparison – ALD Timepoint 100 vs Day 50
Chain Length
Number of Double Bonds
|Log2 Fold Change|
Log2 Fold Change
C
Comparison – ALD Timepoint 150 vs Day 50
Chain Length
Number of Double Bonds
Log2 Fold Change
|Log2 Fold Change|

D

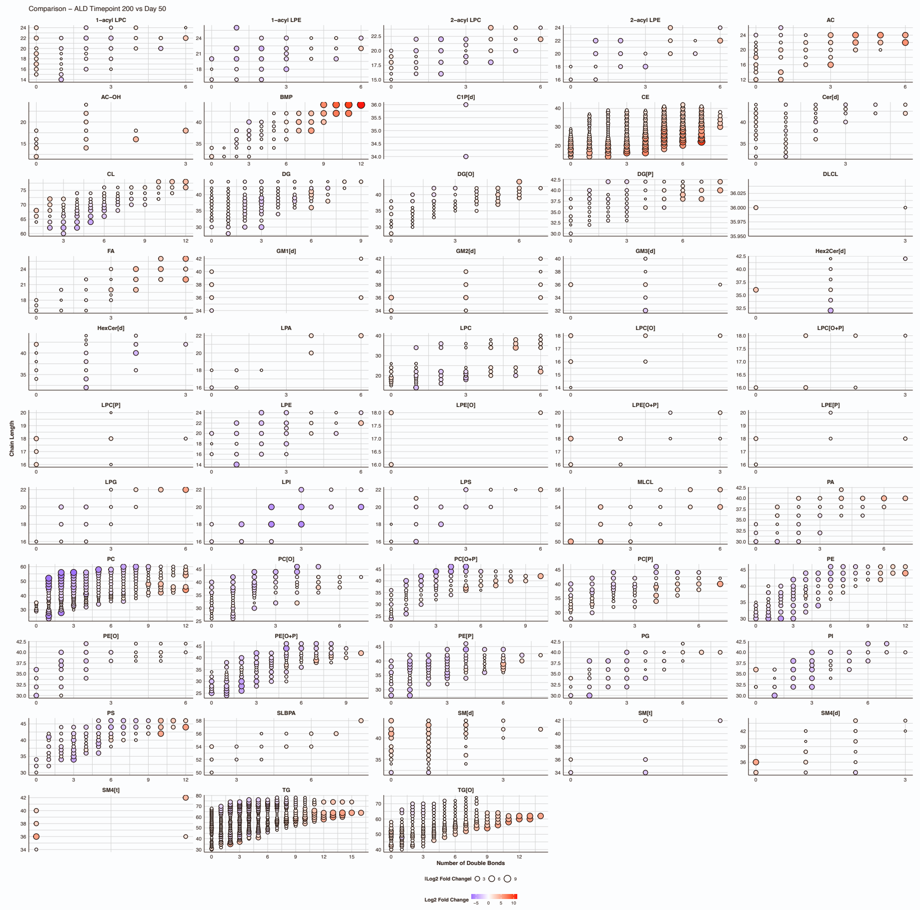

Figure S9

(A) Log2 fold changes of lipid classes with 1, 2, and 3 acyl chains as a function of total number of double bonds across timepoints for ALD hCO (day 100, 150, 200, relative to day 50).

(B) Bubble plots illustrating the relationship between acyl chain length and the number of double bonds for lipid classes in ALD hCO, comparing day 100 to day 50.

(C) Bubble plots illustrating the relationship between acyl chain length and the number of double bonds for lipid classes in ALD hCO, comparing day 150 to day 50.

(D) Bubble plots illustrating the relationship between acyl chain length and the number of double bonds for lipid classes in ALD hCO, comparing day 200 to day 50. Bubble size corresponds to -Log10(p-value) and color intensity indicates Log2 fold change.

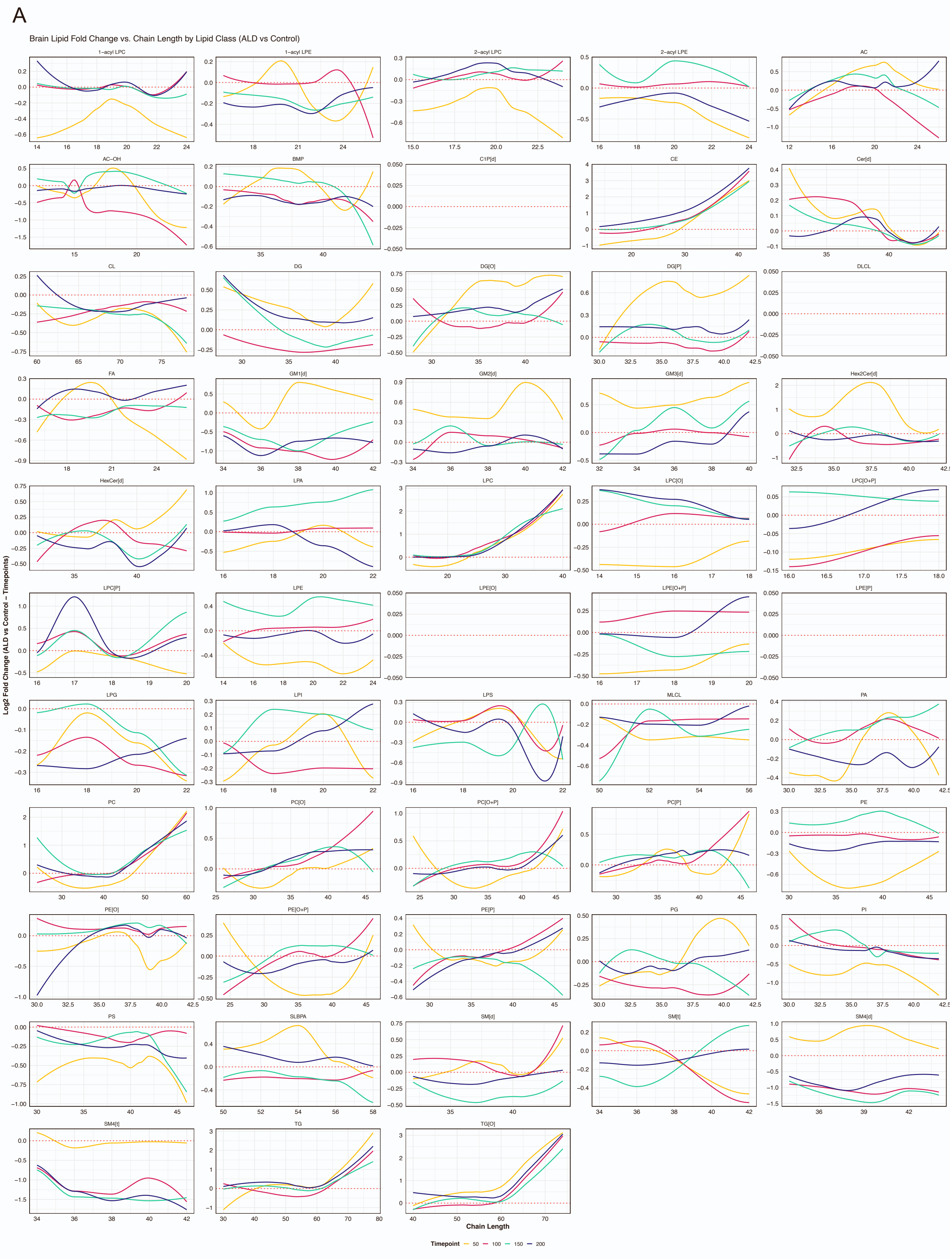
A
Brain Lipid Fold Change vs. Chain Length by Lipid Class (ALD vs Control)
Log2 Fold Change (ALD vs Control – Timepoints)
Chain Length
Timepoint 50 100 150 200
1-acyl LPC
1-acyl LPE
2-acyl LPC
2-acyl LPE
AC
AC-OH
BMP
C1P[d]
CE
Cer[d]
CL
DG
DG[O]
DG[P]
DLCL
FA
GM1[d]
GM2[d]
GM3[d]
Hex2Cer[d]
HexCer[d]
LPA
LPC
LPC[O]
LPC[O+P]
LPC[P]
LPE
LPE[O]
LPE[O+P]
LPE[P]
LPG
LPI
LPS
MLCL
PA
PC
PC[O]
PC[O+P]
PC[P]
PE
PE[O]
PE[O+P]
PE[P]
PG
PI
PS
SLBPA
SM[d]
SM[t]
SM4[d]
SM4[t]
TG
TG[O]

Figure S10

(A) Line plots showing Log2 fold change for lipid classes a function of acyl chain length, comparing ALD to controls. Trends are displayed for Days 50, 100, 150, and 200.

A

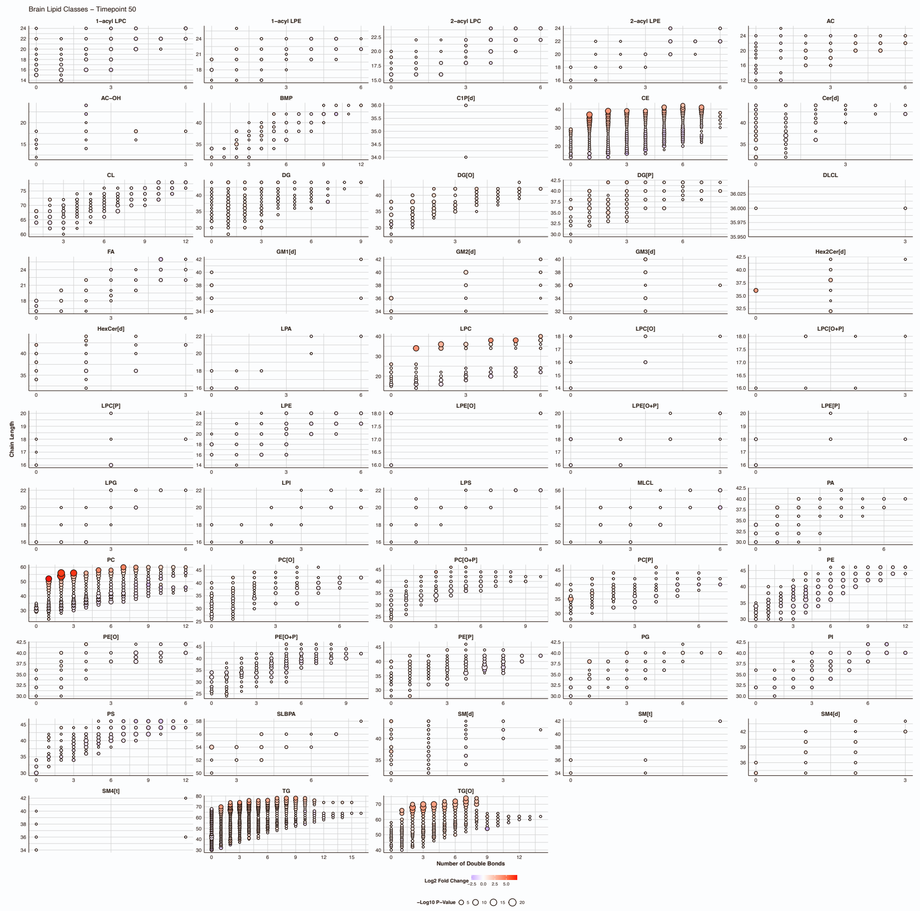
Brain Lipid Classes – Timepoint 50
1−acyl LPC
1−acyl LPE
2−acyl LPC
2−acyl LPE
AC
AC−OH
BMP
C1P[d]
CE
Cer[d]
CL
DG
DG[O]
DG[P]
DLCL
FA
GM1[d]
GM2[d]
GM3[d]
Hex2Cer[d]
HexCer[d]
LPA
LPC
LPC[O]
LPC[O+P]
LPC[P]
LPE
LPE[O]
LPE[O+P]
LPE[P]
LPG
LPI
LPS
MLCL
PA
PC
PC[O]
PC[O+P]
PC[P]
PE
PE[O]
PE[O+P]
PE[P]
PG
PI
PS
SLBPA
SM[d]
SM[t]
SM4[d]
SM4[t]
TG
TG[O]
Chain Length
Number of Double Bonds
Log2 Fold Change
−Log10 P−Value

B

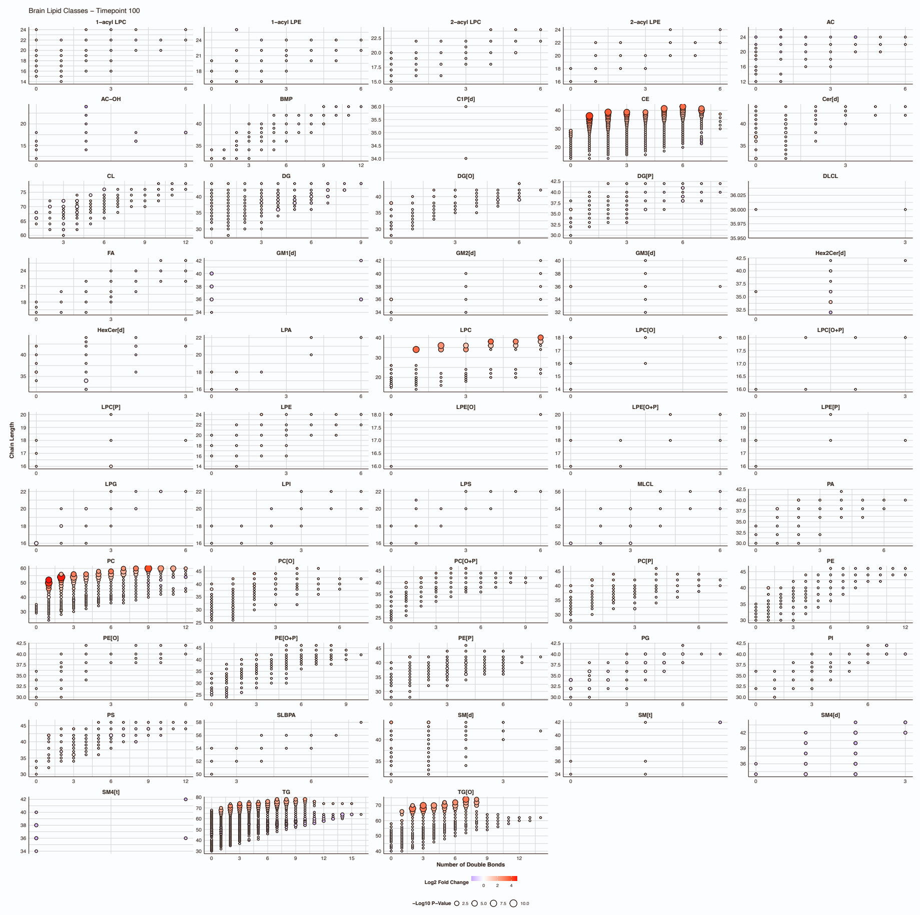
Brain Lipid Classes – Timepoint 100
1−acyl LPC
1−acyl LPE
2−acyl LPC
2−acyl LPE
AC
AC−OH
BMP
C1P[d]
CE
Cer[d]
CL
DG
DG[O]
DG[P]
DLCL
FA
GM1[d]
GM2[d]
GM3[d]
Hex2Cer[d]
HexCer[d]
LPA
LPC
LPC[O]
LPC[O+P]
LPC[P]
LPE
LPE[O]
LPE[O+P]
LPE[P]
LPG
LPI
LPS
MLCL
PA
PC
PC[O]
PC[O+P]
PC[P]
PE
PE[O]
PE[O+P]
PE[P]
PG
PI
PS
SLBPA
SM[d]
SM[t]
SM4[d]
SM4[t]
TG
TG[O]
Chain Length
Number of Double Bonds
Log2 Fold Change
−Log10 P−Value

C

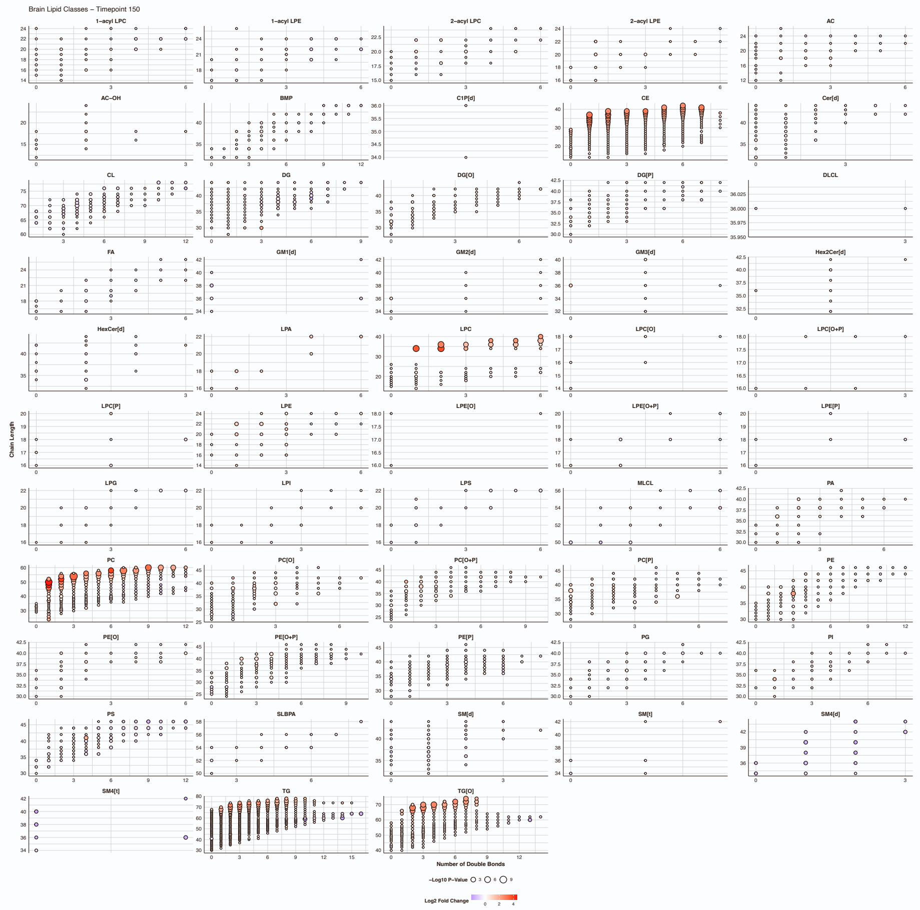

Brain Lipid Classes – Timepoint 150
Chain Length
Number of Double Bonds
-Log10 P-Value
Log2 Fold Change

D

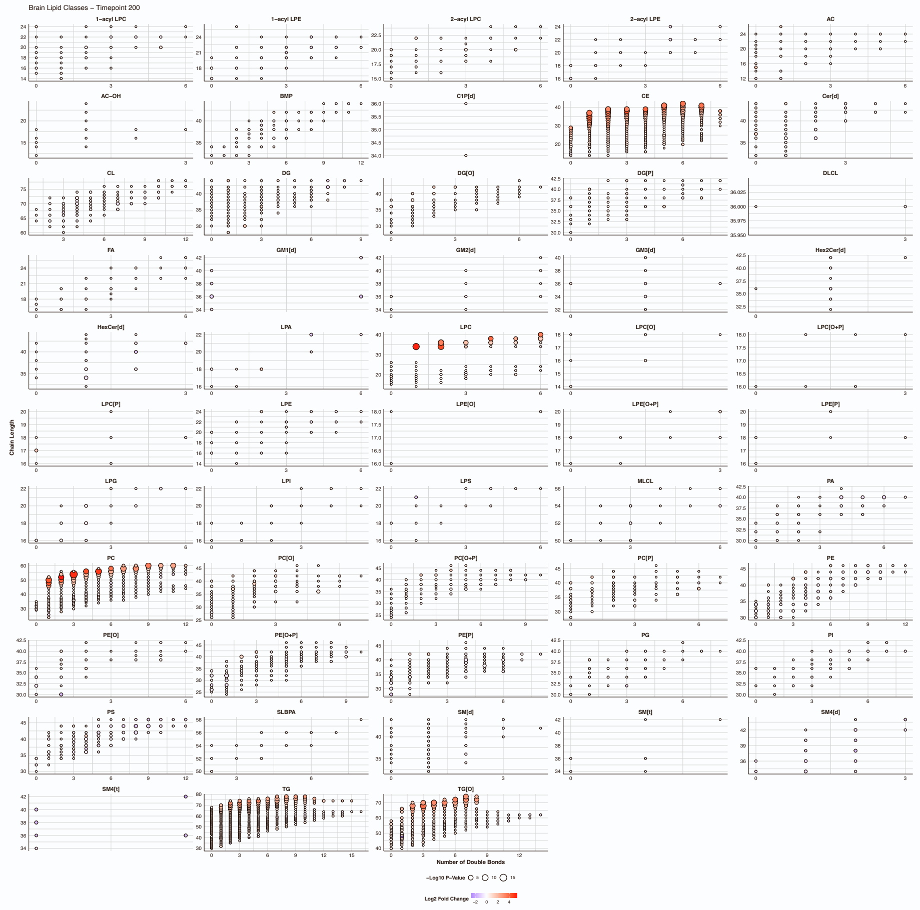

Brain Lipid Classes – Timepoint 200
Chain Length
Number of Double Bonds
-Log10 P-Value
Log2 Fold Change

Figure S11

(A) Bubble plots illustrating the relationship between acyl chain length and the number of double bonds for lipid classes in hCO at day 50 comparing ALD to control.

(B) Bubble plots illustrating the relationship between acyl chain length and the number of double bonds for lipid classes in hCO at day 100 comparing ALD to control.

(C) Bubble plots illustrating the relationship between acyl chain length and the number of double bonds for lipid classes in hCO at day 150 comparing ALD to control.

(D) Bubble plots illustrating the relationship between acyl chain length and the number of double bonds for lipid classes in hCO at day 200 comparing ALD to control. Bubble size corresponds to -Log10(p-value) and color intensity indicates Log2 fold change.

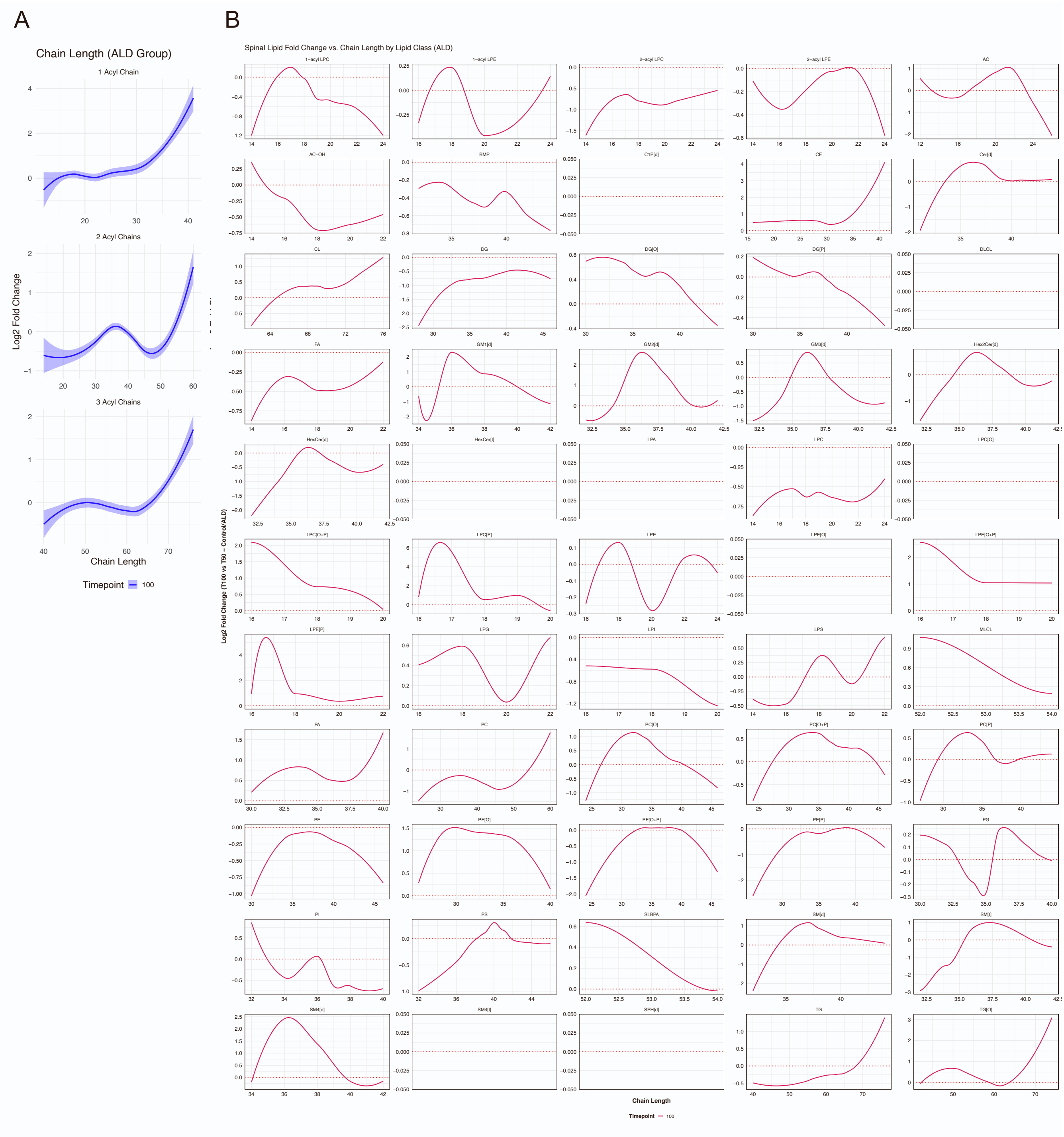

Figure S12

(A) Log2 fold changes of lipid classes with 1, 2, and 3 acyl chains as a function of total acyl chain length across timepoints for ALD hSO (day 100 relative to day 50). **(B)** Log2 fold changes of lipid classes as a function of total acyl chain length of ALD hSO day 100 relative to day 50.

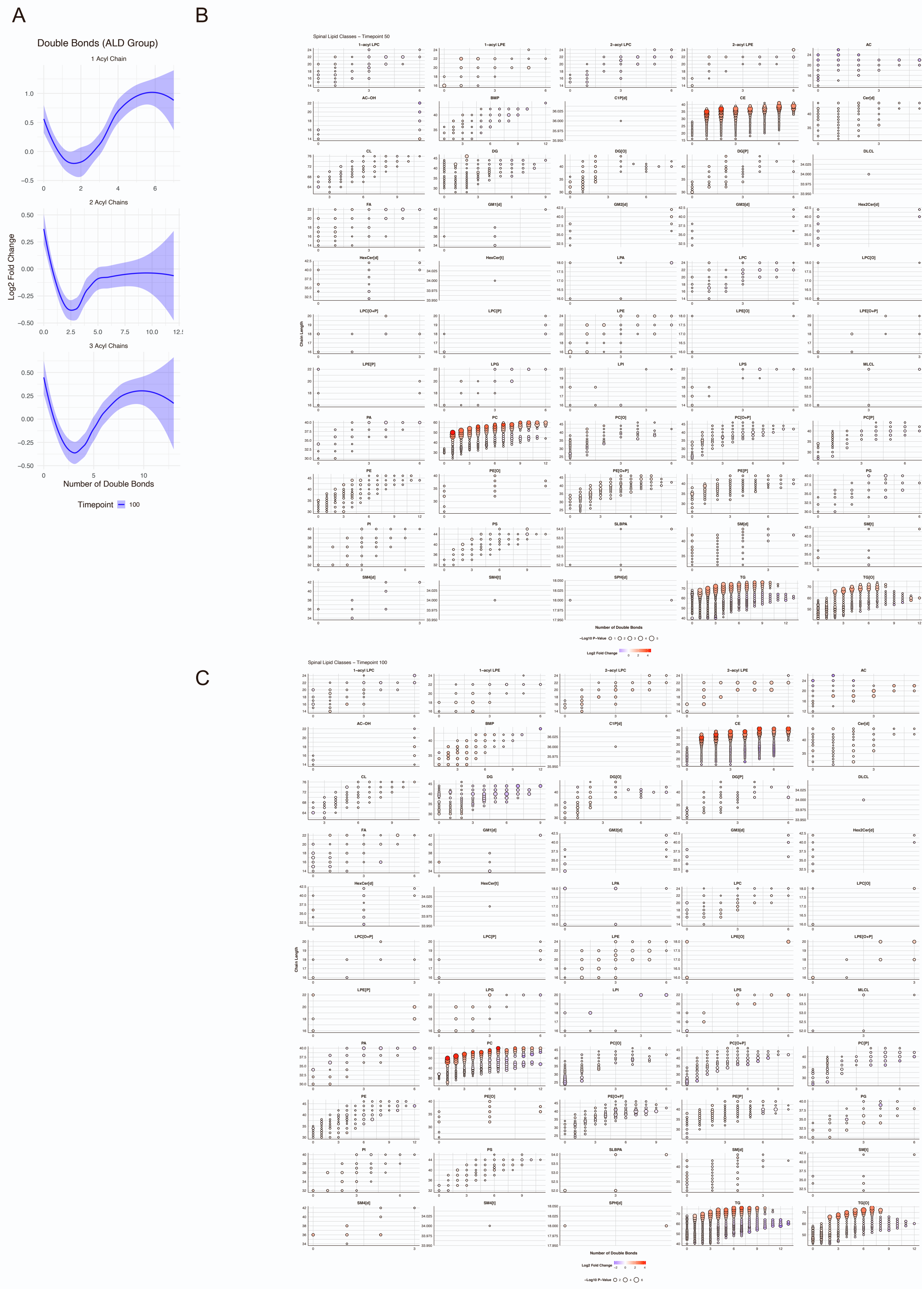
A
B
C
Double Bonds (ALD Group)
1 Acyl Chain
2 Acyl Chains
3 Acyl Chains
Log2 Fold Change
Number of Double Bonds
Timepoint 100
Spinal Lipid Classes – Timepoint 50
Spinal Lipid Classes – Timepoint 100
Chain Length
Number of Double Bonds
–Log10 P–Value
Log2 Fold Change

Figure S13

(A) Log2 fold changes of lipid classes with 1, 2, and 3 acyl chains as a function of total number of double bonds across timepoints for ALD hSO (day 100 relative to day 50).

(B) Bubble plots illustrating the relationship between acyl chain length and the number of double bonds for lipid classes hSO at day 50, comparing ALD to control.

(C) Bubble plots illustrating the relationship between acyl chain length and the number of double bonds for lipid classes hSO at day 100, comparing ALD to control. Bubble size corresponds to -Log10(p-value) and color intensity indicates Log2 fold change.

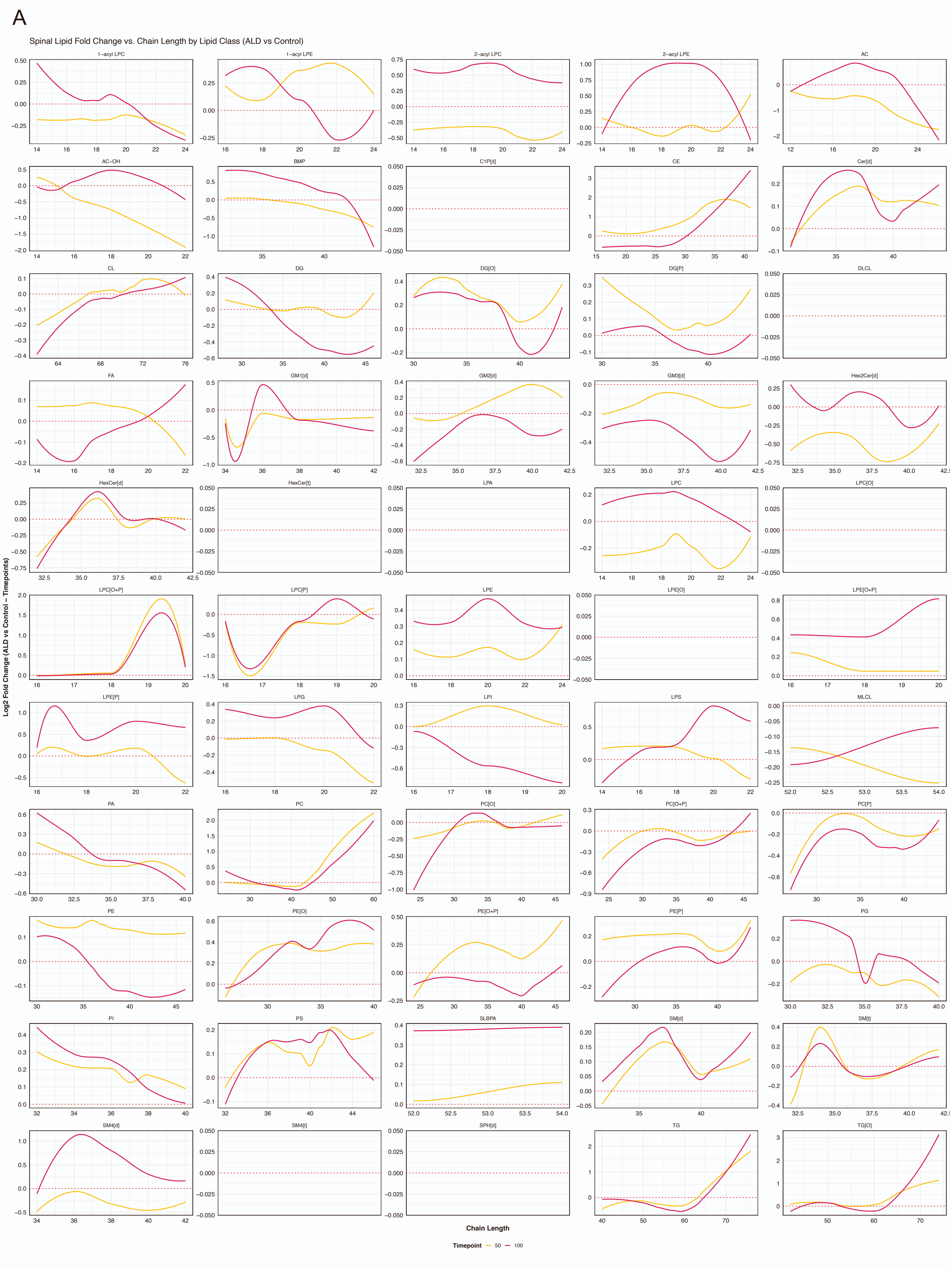
A
Spinal Lipid Fold Change vs. Chain Length by Lipid Class (ALD vs Control)
1−acyl LPC
1−acyl LPE
2−acyl LPC
2−acyl LPE
AC
AC−OH
BMP
C1P[d]
CE
Cer[d]
CL
DG
DG[O]
DG[P]
DLCL
FA
GM1[d]
GM2[d]
GM3[d]
Hex2Cer[d]
HexCer[d]
HexCer[t]
LPA
LPC
LPC[O]
LPC[O+P]
LPC[P]
LPE
LPE[O]
LPE[O+P]
LPE[P]
LPG
LPI
LPS
MLCL
PA
PC
PC[O]
PC[O+P]
PC[P]
PE
PE[O]
PE[O+P]
PE[P]
PG
PI
PS
SLBPA
SM[d]
SM[t]
SM4[d]
SM4[t]
SPH[d]
TG
TG[O]
Log2 Fold Change (ALD vs Control − Timepoints)
Chain Length
Timepoint 50 100

Figure S14

(A) Line plots showing Log2 fold change for lipid classes a function of acyl chain length, comparing hSO ALD to hSO controls. Trends are displayed for Days 50 and 100.

A

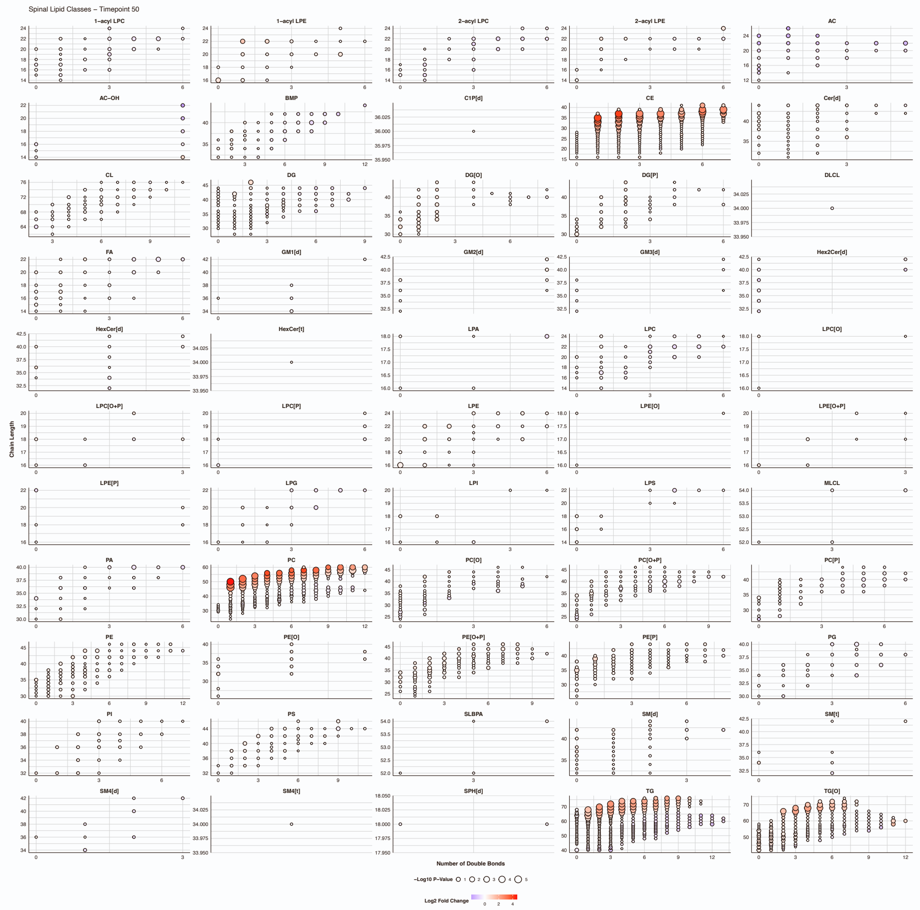
Spinal Lipid Classes – Timepoint 50
Chain Length
Number of Double Bonds
–Log10 P–Value
Log2 Fold Change

B

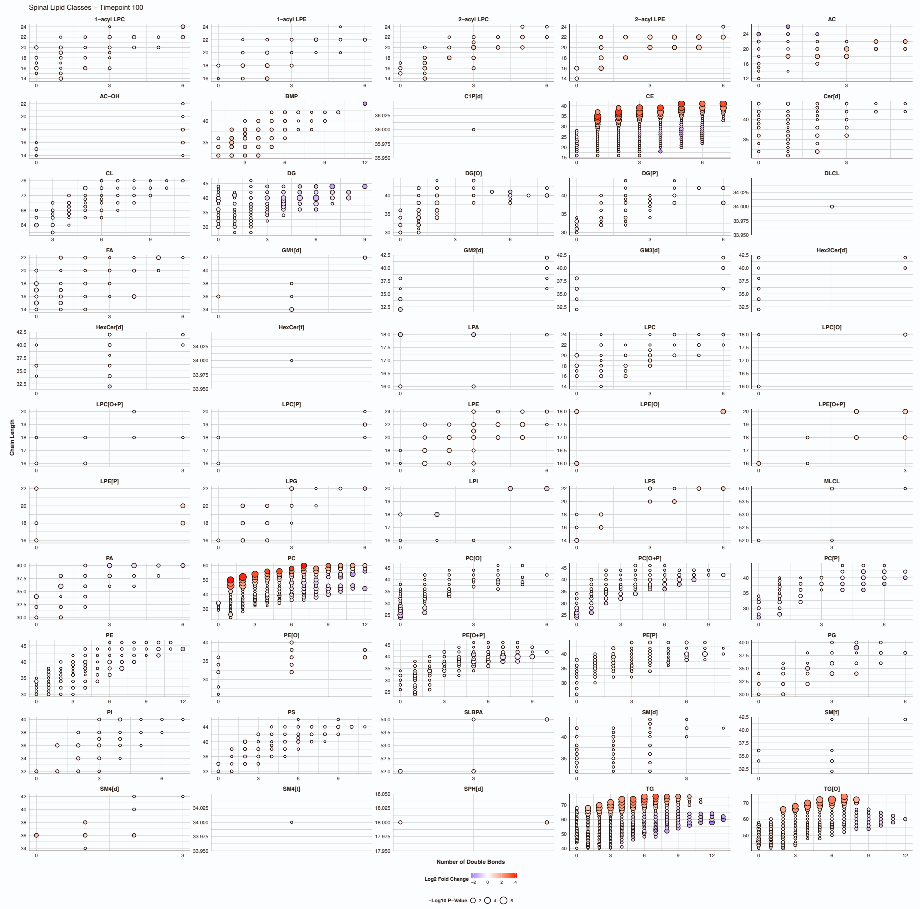
Spinal Lipid Classes – Timepoint 100
Chain Length
Number of Double Bonds
Log2 Fold Change
–Log10 P–Value

Figure S15

(A) Bubble plots illustrating the relationship between acyl chain length and the number of double bonds for lipid classes in hSO at day 50 comparing ALD to control.

(B) Bubble plots illustrating the relationship between acyl chain length and the number of double bonds for lipid classes in hSO at day 100 comparing ALD to control.

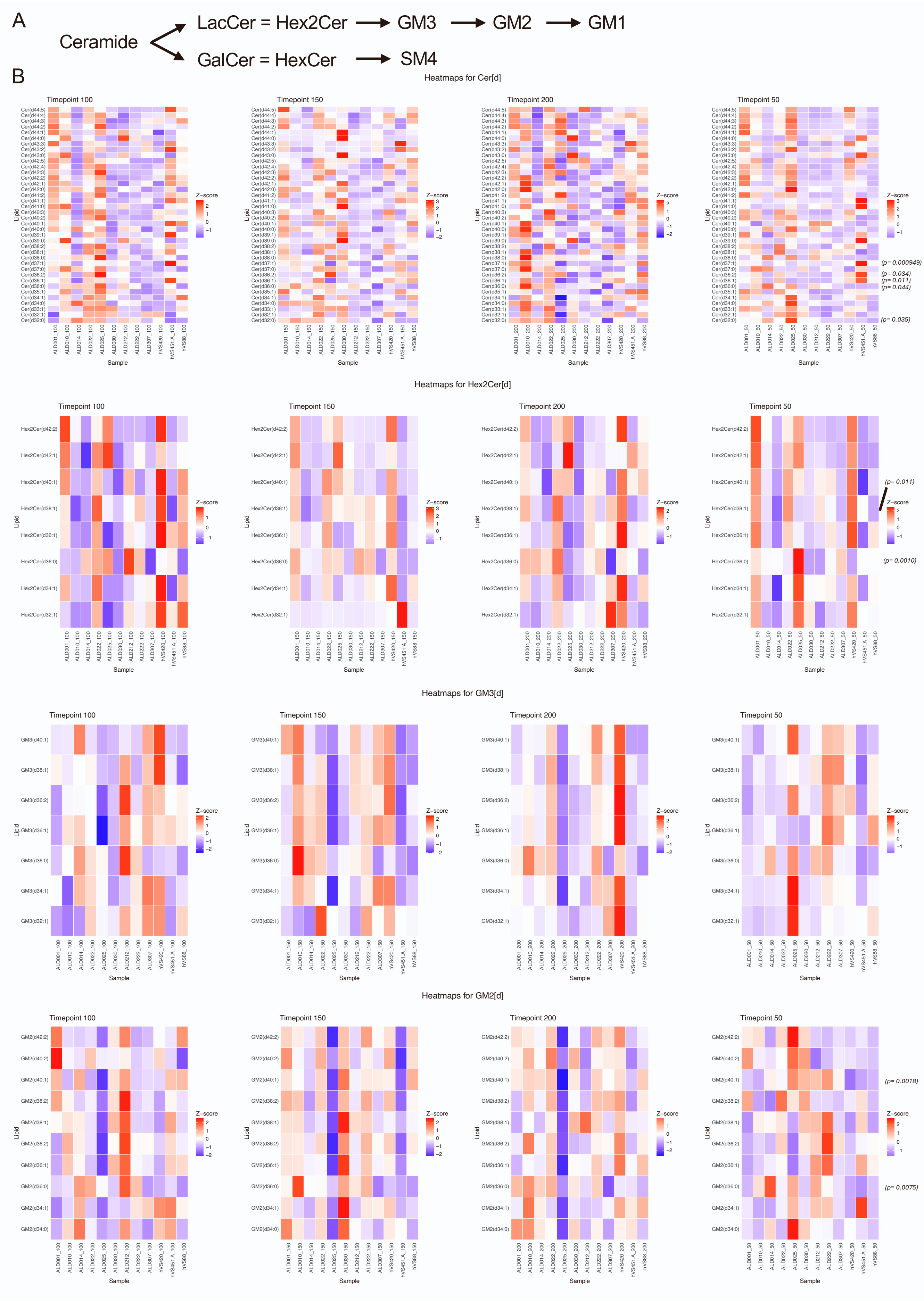
A
Ceramide
LacCer = Hex2Cer
GM3
GM2
GM1
GalCer = HexCer
SM4
B
Heatmaps for Cer[d]
Timepoint 100
Timepoint 150
Timepoint 200
Timepoint 50
(p= 0.000949)
(p= 0.034)
(p= 0.011)
(p= 0.044)
(p= 0.035)
Heatmaps for Hex2Cer[d]
(p= 0.011)
(p= 0.0010)
Heatmaps for GM3[d]
Heatmaps for GM2[d]
(p= 0.0018)
(p= 0.0075)
Z-score
Lipid
Sample

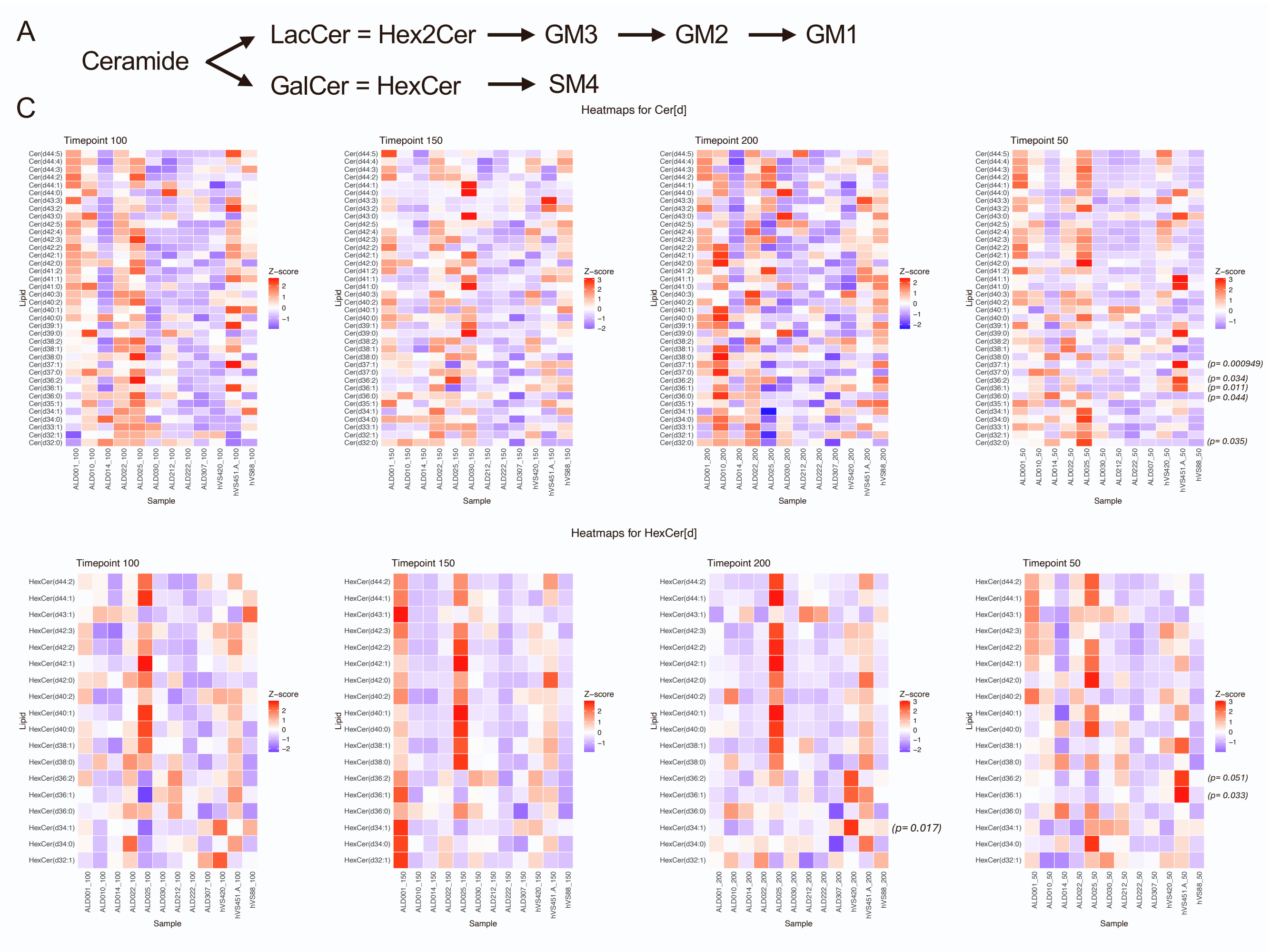

Figure S16

(A) Scheme of the production of gangliosides and sulfatides from ceramides

(B-C) Heatmaps of Z-scores for individual lipid species across samples from ALD and control hCO. Each row represents a lipid species, and each column represents an hiPSC line.

Table S1. hiPSC lines

Cell line	Sex	Age	Phenotype	Mutation	Reprogramming method
Control 1 – hvs88	Male	74 days	Control	No	Lentivirus
Control 2 – hvs420	Male	21 years	Control	No	Lentivirus
Control 3 – hvs451	Male	19 years	Control	No	Sendai virus
ALD1 – ALD001	Male	78 years	ALD	c.901-5C>A	Sendai virus
ALD2 – ALD010	Male	34 years	ALD	c.1390C>T	Lentivirus
ALD3 – ALD014	Male	76 years	ALD	c.659T>C	Lentivirus
ALD4 – ALD022	Male	74 years	ALD	c.1A>G	Sendai virus
ALD 5 – ALD025	Male	21 years	ALD	c.1866-10G>A	Lentivirus
ALD6 – ALD030	Male	65 years	ALD	c.1A>G	Sendai virus
ALD7 – ALD212	Male	40 years	ALD	c.580G>C	Lentivirus
ALD 8 – ALD222	Male	27 years	ALD	c.446G>A	Lentivirus
ALD9 – ALD307	Male	30 years	ALD	c.346G>A	Lentivirus
ALD10 – ALD202	Male	56 years	ALD	c.1166G>A	Sendai virus
